# Pneumococcal vaccine schedules (PVS) study: a cluster-randomised, non-inferiority trial of an alternative versus standard schedule for pneumococcal conjugate vaccination—statistical analysis plan

**DOI:** 10.1186/s13063-022-06900-x

**Published:** 2022-12-28

**Authors:** Grant A. Mackenzie, Arto A. Palmu, Jukka Jokinen, Isaac Osei, Stefan Flasche, Brian Greenwood, Kim Mulholland, Cattram Nguyen

**Affiliations:** 1 MRC Unit The Gambia at London School of Hygiene & Tropical Medicine, Fajara, The Gambia; 2 Faculty of Infectious & Tropical Diseases, London School of Hygiene & Tropical Medicine, London, UK; 3 Infection & Immunity Theme, Murdoch Children’s Research Institute, Melbourne, Australia; 4 Department of Paediatrics, University of Melbourne, Melbourne, Australia; 5 Finnish Institute for Health and Welfare, Helsinki, Finland; 6 Faculty of Epidemiology & Public Health, London School of Hygiene & Tropical Medicine, London, UK

**Keywords:** Pneumococcal, Vaccine, Schedule, Cluster-randomised, Statistical analysis plan

## Abstract

**Rationale:**

The effectiveness of universal immunisation with pneumococcal conjugate vaccine (PCV) has been evident in many countries. However, the global impact of PCV is limited by its cost, which has prevented its introduction in several countries. Reducing the cost of PCV programmes may facilitate vaccine introduction in some countries and improve the sustainability of PCV in EPIs in low-income countries when they transition away from subsidised vaccine supply.

**Methods and design:**

PVS is a real-world field trial of an alternative schedule of one dose of PCV scheduled at age 6 weeks with a booster dose at age 9 months (i.e. the alternative ‘1+1’ schedule) compared to the standard schedule of three primary doses scheduled at 6, 10, and 14 weeks of age (i.e. the standard ‘3+0’ schedule). Delivery of the interventions began in late 2019 in 68 geographic clusters and will continue for 4 years. The primary endpoint is the prevalence of nasopharyngeal vaccine-type pneumococcal carriage in children aged 2–260 weeks with clinical pneumonia in year 4. Secondary endpoints are the prevalence of vaccine-type pneumococcal carriage among all ages in year 4 and the incidence of radiological pneumonia in children enrolled to receive the interventions. Additional disease and carriage endpoints are included.

**Purpose:**

This statistical analysis plan (SAP) describes the cohorts and populations, and follow-up criteria, to be used in different analyses. The SAP defines the endpoints and describes how adherence to the interventions will be presented. We describe how analyses will account for the effect of clustering and stratified randomisation. The SAP defines the approach to non-inferiority and other analyses. Defining the SAP early in the trial will avoid bias in analyses that may arise from prior knowledge of trial findings.

**Supplementary Information:**

The online version contains supplementary material available at 10.1186/s13063-022-06900-x.

## Introduction

This statistical analysis plan follows the reporting guidelines described by Rodriguez et al. [1] and the UK Clinical Research Collaboration Registered Clinical Trial Unit Statisticians’ Operational Group [2], as well as CONSORT guidelines for reporting cluster-randomised [3] and non-inferiority [4] trials. The trial protocol is available on the *Trials* website [5].

The following terminology is used to refer to the analysis populations/cohorts and types of analyses:

- Individual-level cohort—participants enrolled as infants at Expanded Programme on Immunisation (EPI) clinics to receive the trial interventions
- Individual-level analysis—approach to regression analysis of cluster-randomised trials including data on individual participants while also accounting for cluster-level correlation
- Cluster-level analysis—approach to regression analysis of cluster-randomised trials that summarises data at the cluster-level which is then used in a further step to estimate the intervention effect
- Population-level population/cohort—all resident children, assigned to each group according to village of residence, under surveillance for endpoints, and included in analysis regardless of enrolment as an infant to receive the study intervention

### Background and rationale

The Gambian Expanded Programme on Immunisation (EPI) introduced the 7-valent pneumococcal conjugate vaccine (PCV7) in 2009 and PCV13 in 2011, using the standard schedule of three doses in early infancy. Compared to the pre-vaccine period, in 2016–2017, there was a 92% reduction in the incidence of vaccine-type (VT) invasive pneumococcal disease (IPD) in the 2–59-month age group [6]. PCV also prevents the acquisition of VT pneumococcal carriage, with resulting indirect herd protection effects in the population. However, the prevalence of nasopharyngeal (NP) carriage of VT pneumococci in young children remains around 15% (author’s own data), having fallen from 47% in the pre-vaccine period [7]. The global impact of PCV is limited by its cost, which has prevented its introduction in several countries. Reducing the cost of PCV programmes may facilitate vaccine introduction in some countries and improve the sustainability of EPI programmes in low-income countries when they transition away from subsidised vaccine supply through Gavi, the Vaccine Alliance.

This pragmatic cluster-randomised, non-inferiority field trial is being conducted in the Basse and Fuladu West Health and Demographic Surveillance Systems (BHDSS and FWHDSS) in rural Gambia. Cluster-wise delivery of the schedules at EPI clinics over 4 years in distinct geographic populations will allow direct and indirect effects of the schedules on pneumococcal transmission and disease to develop within each geographic population cluster. Because the incidence of IPD and radiological pneumonia has fallen greatly with PCV vaccination, such endpoints cannot be primary endpoints in this trial. We have chosen NP carriage of VT pneumococci in children with clinical pneumonia as the primary endpoint. Disease measures are included, primarily related to safety. We also include a number of additional pneumococcal carriage endpoints at the community-level.

Using the framework of Halloran et al., for study designs to measure different effects of vaccines and vaccination [8], this trial aims to estimate and compare the overall public health effects of the two PCV schedules. Overall public health effects include the indirect effects of widespread vaccination on unvaccinated individuals [8]. The combination of direct and indirect effects of widespread vaccination on vaccinated individuals is termed the total effects of vaccination and vaccination programme. The overall public health effects of the vaccination programme depend on the weighted average of indirect effects on unvaccinated individuals and the total effects on vaccinated individuals [8].

The primary endpoint analysis will use the population-level population with observations in year 4 after the development of the direct and indirect effects of the schedules (Table 1). All children aged 2–260 weeks with clinical pneumonia in year 4, and resident in the trial area, whether enrolled to receive trial interventions or not, will be included in the analysis. The analysis will provide information combining the direct and indirect effects of the PCV schedules on VT pneumococcal transmission with interpretation related to the individual resident child.

**Table 1:** Analysis framework for populations/cohorts, observations/follow-up, and outcome measures

Endpoint Designation	Population/cohort	Observation/follow-up	Endpoint	Effect measure
Primary	^a^Resident children and clinical pneumonia	Continuous surveillance	^b^VT carriage	Prevalence ratio
Secondary	^c^Enrolled children	^d^Intention-to-treat	Radiological pneumonia	Incidence ratio
	All resident population	Cross-sectional	^e^VT carriage	Prevalence ratio
Tertiary	^a^Resident children and clinical pneumonia	Continuous surveillance	^f^NVT and ^g^Spn carriage	Prevalence ratio
	Age 6–12 weeks no PCV	Cross-sectional	^h^VT carriage	Prevalence ratio
	^c^Enrolled children and clinical pneumonia	^d^Intention-to-treat	VT, NVT, and ^g^Spn carriage	Prevalence ratio
	^i^Enrolled children received intervention and clinical pneumonia	^§§^Per-protocol	VT, NVT, and ^g^Spn carriage	Prevalence ratio
	Resident children	Continuous surveillance	VT, NVT, and ^g^Spn IPD	Incidence ratio
	“	“	Radiological pneumonia	“
	“	“	Clinical pneumonia	“
	“	“	Clinical pneumonia and VT carriage	“
	“	“	Hypoxic pneumonia	“
	“	“	Hospitalisation	“
	“	“	Mortality	“
	Enrolled children	^d^Intention-to-treat	VT, NVT, and ^g^Spn IPD	“
	“	“	Clinical pneumonia	“
	“	“	Clinical pneumonia and VT carriage	“
	“	“	Hypoxic pneumonia	“
	“	“	Hospitalisation	“
	“	“	Mortality	
	Enrolled children received intervention	^j^Per-protocol	Radiological pneumonia	“
	“	“	VT, NVT, and Spn IPD	“
	“	“	Clinical pneumonia	“
	“	“	Clinical pneumonia and VT carriage	“
	“	“	Hypoxic pneumonia	“
	“	“	Hospitalisation	“
	“	“	Mortality	“

^a^Population-based population aged 2–260 weeks. ^b^Primary endpoint specified in year 4 and also calculated as tertiary endpoints in years 1, 2, and 3. ^c^Cohort enrolled to receive interventions. ^d^Events counted from enrolment to end of follow-up. ^e^Secondary endpoint specified in year 4 and also calculated as a tertiary endpoint in year 3 (all ages and age-stratified). ^f^Calculated in years 1, 2, 3, and 4. ^g^Spn = all pneumococcal carriage. ^h^In year 4. ^i^Cohort enrolled and received interventions PP. ^j^Events counted from 14 days post-PCV1 to end of follow-up

The trial includes two secondary endpoints (Table 1). The secondary endpoint of radiological pneumonia incidence will include all children enrolled to receive the interventions and function primarily as a safety endpoint. This individual-level cohort will experience the direct effects of the different PCV schedules and their increasing indirect effects over 4 years. The radiological pneumonia endpoint offers reasonable specificity [9] to detect a potential difference in the incidence of this important disease endpoint. However, limited frequency and statistical power precludes specification of a non-inferiority hypothesis for this endpoint. We will present a point estimate and confidence interval indicating the potential range of values of the incidence rate ratio of radiological pneumonia in the two groups. An additional secondary endpoint will be VT pneumococcal carriage at the community-level in all ages in year 4. This population-level measurement of VT carriage will include direct and indirect effects after 4 years of intervention delivery in the population.

Tertiary endpoints will include year 4 measurement of VT carriage in 6–12-week-old infants who have not yet been immunised with PCV. This analysis will relate only to indirect effects in this important age group. Endpoints specifying non-vaccine type (NVT) pneumococcal carriage will compare the effects of the schedules on potential ‘serotype replacement’ in carriage that follows vaccine-induced reductions in VT carriage. Additional disease endpoints will further inform the safety of the alternative schedule (Table 1). Specification of multiple complementary endpoints means that overall interpretation of the trial results will require synthesis of all the available information.

Analyses using the population-level population will compare the impact of the schedules relating to the total population, as is appropriate for decision-making in mature immunisation programmes. Analyses will also be conducted in the individual-level cohort enrolled to receive the intervention. Individual-level cohort analyses will provide information on the biological effects of the schedules, particularly related to the relative safety of the two schedules that can most easily be interpreted in the light of the wide range of settings where the schedules could be used. Differences between per-protocol (PP) and intention-to-treat (ITT) analyses of the individual-level cohort will relate mainly to delays of receipt of scheduled doses and incorrect administration of schedules, which will differ from one setting to another.

The setting is a rural African location where over 75% of vaccination is provided through outreach visiting villages only once per month. The age at vaccination may therefore be different from that which applies in urban African settings, where vaccination may be more frequently available. The conduct of the trial has enhanced service delivery for the primary series of doses by a marginal degree so the age at vaccination in the trial is largely representative of the situation that would pertain should the alternative schedule be introduced in the routine EPI. However, the timing and coverage of the PCV booster dose at 9 months of age may have been enhanced to some degree. Thus, ITT analysis using the individual-level cohort will show the impact of the alternative schedule under a particular set of vaccination delivery circumstances that may not represent a routine setting. Analysis in the population-level population most clearly defines the conditions needed to combine all direct and indirect effects of the schedules in the population. The individual-level cohort PP results can most easily be compared between different study sites. The trial will include mathematical modelling of schedule effects on VT carriage with varied coverage of the alternative schedule PCV booster dose. Table 1 presents the analysis framework, specifying the populations and cohorts, observations and follow-up, endpoints, and effect measures.

### Objectives

The trial aims to account for the direct and indirect effects of vaccination with PCV13 at the population-level. We aim to test whether transition from the standard schedule to an alternative schedule, with doses scheduled at 6 weeks and 9 months of age, is non-inferior to continued use of the standard scheduling of doses at 6, 10, and 14 weeks of age.

#### Endpoints

Primary endpoint

- Prevalence of NP carriage of VT pneumococci in children aged 2–260 weeks with clinical pneumonia in year 4

Secondary endpoints

- Incidence of radiological pneumonia in children enrolled to receive intervention
- Population-based NP carriage prevalence of VT pneumococci year 4 (tertiary endpoint year 3)

Tertiary endpoints

- Prevalence of NP carriage of NVT and all pneumococci in children aged 2–260 weeks with clinical pneumonia in year 4
- Prevalence of NP carriage of VT, NVT, and all pneumococci in unimmunised infants aged 6–12 weeks in year 4
- Prevalence of NP carriage of VT, NVT, and all pneumococci in enrolled children (and children who received intervention) with clinical pneumonia
- Incidence of VT, NVT, and all IPD in resident children aged 2–260 weeks
- Incidence of radiological pneumonia in resident children aged 2–260 weeks
- Incidence of clinical pneumonia in resident children aged 2–260 weeks
- Incidence of clinical pneumonia associated with NP carriage of VT pneumococci in resident children aged 2–260 weeks
- Incidence of hypoxic pneumonia in resident children aged 2–260 weeks
- Incidence of hospitalisation in resident children aged 2–260 weeks
- Incidence of mortality in resident children aged 2–260 weeks
- Incidence of VT, NVT, and all IPD in enrolled children and enrolled children who received intervention
- Incidence of radiological pneumonia in enrolled children and enrolled children who received intervention
- Incidence of clinical pneumonia in enrolled children and enrolled children who received intervention
- Incidence of clinical pneumonia associated with NP carriage of VT pneumococci in enrolled children and enrolled children who received intervention
- Incidence of hypoxic pneumonia in enrolled children and enrolled children who received intervention
- Incidence of hospitalisation in enrolled children and enrolled children who received intervention
- Incidence of mortality in enrolled children and enrolled children who received intervention

#### Other analyses

The first endpoint below aims to detect potential early impact of the alternative schedule:

- Prevalence of NP carriage of VT pneumococci in children aged 2–260 weeks with clinical pneumonia in year 2
- Incidence of non-pneumococcal invasive bacterial disease, presentations with diarrhoea, presentations with cough or cold/upper respiratory tract infection, hospitalisations with diarrhoea, and proportion of clinical pneumonia patients who are hospitalised (analyses to detect potential differential case ascertainment or access to care)

## Study methods

### Trial design

This is a cluster-randomised, non-inferiority, parallel-group, unmasked field trial under real-world conditions. This rural Gambian population is the same as that included in an earlier trial that established the efficacy of PCV9 [9] and effectiveness of routine infant vaccination with PCV13 [6, 10, 11]. The schedule tested in the PCV9 trial (doses scheduled at 6, 10, 14 weeks of age) is the same as the standard schedule in this non-inferiority trial. Efficacy of PCV9 was established using the endpoint of radiological pneumonia while the primary endpoint in this trial is VT carriage prevalence in children with clinical pneumonia. This trial includes radiological pneumonia as a secondary (safety) endpoint.

#### Definition of cluster and design application to clusters

The trial area of the BHDSS and FWHDSS consists of 68 contiguous geographic units within which resident infants are assigned to attend the one EPI clinic in that geographic area. All the geographic clusters in the BHDSS and FWHDSS were eligible for the trial. The geographic clusters of villages were mapped and designed to minimise the interaction of children in different clusters through school attendance. The average cluster population is 4000, although with significant variability. Group allocation of infants enrolled at EPI clinics is determined by the allocation of the village of residence.

#### Trial design

Each cluster is randomly allocated, in a 1:1 ratio, to one of two study groups. Infants who have not completed their PCV schedule and are aged <9 months are allocated to receive either the alternative or standard PCV schedule. The alternative schedule specifies eligibility for doses at 6 weeks and 9 months of age. The standard schedule specifies eligibility for doses at 6, 10, and 14 weeks of age. This design permits the estimation of the combined direct and indirect effects at the population-level.

### Randomisation

An independent statistician prepared the randomisation lists. All clusters were randomised at the beginning of the trial using a blocked scheme to ensure similar numbers of clusters were assigned to each group. Randomisation was stratified by (1) location (BHDSS or FWHDSS) and (2) a binary variable of ‘high’ or ‘low’ cluster-level incidence of clinical pneumonia (which correlates with VT pneumococcal carriage prevalence). Randomisation was carried out using the above stratification until restricted selections were achieved in which Basse and Bansang towns were in either trial group (the only two hospitals in the trial area are in Basse and Bansang), there was a balance in terms of the presence of a health facility in allocated clusters (i.e. six in one group and five in the other group), and there was balance on total population size between the two groups (i.e. <10% difference in the two groups). Random allocation was performed and revealed in a public event in which one of 100 valid randomisation lists was randomly selected.

All resident infants are eligible for enrolment in the trial, according to the cluster allocation of their village of residence. Consent is sought for the enrolment of infants at EPI clinics to receive the interventions. Consent is also sought for the investigation of patients presenting to health facilities who are under surveillance for clinical endpoints, and also from pneumococcal carriage survey participants.

### Sample size

#### Non-inferiority margin

We determined the non-inferiority margin using the concept of ‘largest loss of impact of the current treatment that would be clinically acceptable’, an approach recommended by the US FDA [12]. The non-inferiority margin uses a relative metric as the relative effect of the standard schedule is likely to be constant [13]. The introduction of the standard schedule has reduced NP VT prevalence in children from 47 to 13%, i.e. a 72% reduction (author’s own data). Baseline risk may change over time, so an absolute value for the non-inferiority margin may not provide a reliable measure of the difference in impact of the two schedules.

Given the advantages of the alternative schedule, the non-inferiority margin will be a 15% loss of the impact of the standard schedule; this value is supported by empiric survey data. In March 2017, an online survey was sent to 72 individuals involved in pneumococcal vaccine research, policy, and clinical care. Valid responses were received from 19 respondents. The survey question presented 10 hypothetical results of VT prevalence in the two trial groups, on a scale of increasing ‘loss of impact’ associated with the alternative schedule, from a baseline of no loss (i.e. a 0% loss of impact) up to a 50% loss of impact. Respondents were asked to consider themselves as decision-makers in their national immunisation programmes, requested to consider a change to their national programme from the standard to the alternative schedule based on the results of the trial. Respondents selected one option corresponding to the ‘loss of impact’ which would sway their decision against introducing the alternative schedule. Using the metric of a percentage loss of impact of the standard schedule which would sway decisions against the new schedule the mean value was a 22.2% loss of impact. The results of the survey support use of a non-inferiority margin that the alternative schedule be associated with a ≤15% loss of impact compared to the standard schedule.

The prevalence of NP carriage of VT pneumococci in the BHDSS among young children with clinical pneumonia from Jan–Sept 2015 was 17% and fell to 15% in Oct–Dec 2015. Assuming VT prevalence of 13% in the standard schedule group at the end of the trial, a 15% loss of impact translates to prevalence in the alternative schedule group of (1 − [0.72 − (0.72 × 0.15)]) × 47% = 18.3%. The non-inferiority margin is expressed in terms of the prevalence ratio outcome measure as 18.0/13.0= 1.38. We used changes in VT prevalence to inform the non-inferiority margin because reductions in VT carriage following the introduction of PCV can predict the impact of PCV on IPD [14] and because carriage prevalence is proportional to the rate of pneumococcal transmission.

For the secondary endpoints of VT pneumococcal carriage prevalence in population-based surveys and among infants aged 6–12 weeks unimmunised with PCV, we will also use the non-inferiority margin of a ≤15% loss of impact in the alternative compared to standard schedule.

#### Sample size calculation

Sample size calculations were based on the primary outcome of NP carriage of VT pneumococci in children aged 2–260 weeks with clinical pneumonia. Initial sample size calculations assumed an equal number of 60 participants measured for the primary endpoint in each cluster, that is, 2040 in each group and 4080 in total. Using Basse data from 2015, we assumed the prevalence of VT carriage to be 13% in both the standard and alternative schedule groups. The alternative schedule will be considered non-inferior if the upper limit of the two-sided 95% confidence interval for the prevalence ratio is ≤1.38 (0.18/0.13 = 1.38). Using Basse data, we estimated the intra-class correlation coefficient (ICC) to be 0.01–0.02. We calculated study power using the methods of Farrington et al. [15] and Donner et al. [16]. Using the more conservative value for ICC of 0.02, a minimum number measured for the primary endpoint per cluster of 60, with 34 clusters per arm (with design effect of 2.18) and *α*=0.05, a sample size of 2040 per arm will provide 93% power to test the non-inferiority of the alternative schedule.

To increase confidence in the study power, we simulated data using 2015 BHDSS data on cluster-wise VT carriage prevalence in children with pneumonia. The simulated data were designed to be similar to the villages included in this trial with respect to the mean carriage prevalence, as well as the variability in prevalence across clusters. Using 1000 simulated populations, each consisting of 68 clusters of 60 individuals, baseline prevalence of 10% and a largest acceptable increase of 4%, i.e. from 10% to 14% (i.e. non-inferiority prevalence ratio of 0.14/0.10=1.40), study power was ≥85% in most scenarios.

During the piloting of trial procedures, it became clear that the number of patients measured for the NP carriage endpoint would be quite variable among clusters with less than 60 patients in some clusters. Further simulations using local data on the variable numbers expected per cluster in 1 year (*N*=3948, median=33, range 7–506) and assuming VT prevalence of 16% in both groups demonstrated trial power of 90%. In order to maximise trial power, the primary outcome will be measured in all eligible patients in each of the 68 clusters with a total number ≥4080 included in the final year 4 analysis.

In the community carriage surveys in years 3 and 4, we will sample 60 residents in each of the 68 clusters. The same assumptions and methods of power calculation as for the primary endpoint will apply. We assume ICC=0.02, 15% baseline VT carriage prevalence in both groups, and a ≤1.38 prevalence ratio derived from the non-inferiority margin of a ≤15% ‘loss of effect’ compared to the standard schedule. If the upper bound of the 95% confidence interval of our prevalence ratio comparing the alternative to the standard schedule clusters is ≤1.38, then the alternative schedule will be considered non-inferior. Following these assumptions, the community carriage surveys in years 3 and 4 will have power >94% if we collect 4080 samples evenly across 68 clusters (i.e. 60 per cluster).

Power for the tertiary endpoint of VT prevalence in PCV unimmunised infants aged 6–12 weeks (assume VT carriage prevalence of 10%) will be similar to that for the primary endpoint. The study is not powered to determine non-inferiority for other endpoints.

As opposed to the non-inferiority hypothesis that is specified for the primary endpoint in year 4, we will specify a superiority hypothesis for the analysis of year 2 data on VT prevalence in children with clinical pneumonia. Setting a 5% level of significance with a two-sided test, power of 80%, with ICC=0.02, and assuming prevalence of VT carriage in the control group is 22%, we wish to detect an absolute difference of ≥7%. That is, the smallest clinically significant difference that we wish to detect is equivalent to prevalence of 15% versus 22% in the two groups and a prevalence ratio of 15.0/22.0=0.68. Given these parameters, if we measure the endpoint in 25 individuals per cluster, we would need to include 29 clusters in each group, that is, a total of 58 clusters. We will measure the endpoint in all 68 clusters using all the available measurements of the endpoint in year 2.

### Framework

The hypothesis framework is one of non-inferiority with inference at the individual level. A number of endpoints with limited numbers of events will be analysed without a non-inferiority framework and interpretation will be based only on a point estimate and confidence interval for the effect measure. For the analysis of VT pneumococcal carriage in year 2, we specify a superiority framework to test for potential early impact of the alternative schedule.

### Interim analyses and stopping guidance

#### Interim analyses

No interim analyses are planned. Analysis of the endpoint of VT prevalence in children with clinical pneumonia in year 2 will be conducted to provide information for the funding agencies and neither the investigators nor Trial Steering Committee (TSC) will be aware of the results. The Data Monitoring Committee (DMC) will be aware of the results. The aim of the year 2 analysis is to identify potential early differential effects between the two groups. Given that no interim analyses are planned, there are no planned adjustments to the significance level due to interim analyses.

#### Guidance for stopping the trial early

The trial may be stopped early if there is evidence that the risk of pneumococcal disease is greater in one compared to the other trial group. Examples of such findings are group-wise differences in the incidence of VT IPD, radiological pneumonia, or hospitalisation. Although the correlation between the prevalence of VT carriage and disease risk is unclear, and carriage is not a measure of disease risk, the DMC may also gain appreciation of trial safety from the measures of VT carriage in children with clinical pneumonia and in community surveys. Rather than specify a formal stopping rule, the DMC will regularly view the accruing data, considering the overall pattern of results, including the consistency of any effects, the potential for effects to change over time, particularly the accumulation of indirect effects over time [17], and the potential for cluster-specific events of a temporal nature.

If the DMC recommends that the trial be stopped early, a joint meeting of the DMC, TSC, and Central Stakeholder Committee will make a recommendation to the Sponsor regarding post-trial procedures, including whether a dose of PCV be administered to children in a group found to be inferior.

### Timing of analyses

#### Timing of analysis of year 2 data

The analysis of year 2 data on VT prevalence in children with clinical pneumonia will be conducted in year 3, following the approval of the statistical analysis plan (SAP) by the DMC and TSC, and following submission of the SAP for publication. The results of this analysis will be concealed from the investigators and TSC but available to the DMC and funding agencies.

#### Timing of final analyses

Final analyses will be conducted at one point in time but presented in two manuscripts, one including the primary and secondary endpoints and a second including the tertiary endpoints.

### Timing of outcome assessments

#### Timing of primary endpoint assessment

The primary outcome of VT carriage in children with clinical pneumonia is measured continuously throughout the course of the trial among resident children presenting unwell to health facilities in the trial area. That is, the outcome is assessed whenever a resident child presents unwell to a health facility, at any age between 2 and 260 weeks. This outcome will be presented as prevalence at four time points measured using data collected during each of the 4 years of intervention. The primary endpoint will be analysed using data from year 4 of intervention, following the greatest observable accumulation of the effects of the two schedules. Based on the number of patients with clinical pneumonia enrolled after elapse of 11 months in year 4, the 12-month period may be extended by 1–2 months to achieve the required sample size. Table 2 shows the results of the relative impact of the alternative versus standard schedule on VT carriage prevalence in children with clinical pneumonia.

**Table 2:** Results of vaccine schedule impact on the prevalence of vaccine-type pneumococcal carriage in children aged 2–260 weeks with clinical pneumonia

	Alternative schedule			Standard schedule			Adjusted prevalence ratio (95% CI)
	Number VT *Spn*	Number measured	Prevalence % (95% CI)	Number VT *Spn*	Number measured	Prevalence (95% CI)	
Year 1	n	N	n/N ()	n	N	n/N ()	x.y (95% CI)
Year 2	n	N	n/N ()	n	N	n/N ()	x.y (95% CI)
Year 3	n	N	n/N ()	n	N	n/N ()	x.y (95% CI)
Year 4	n	N	n/N ()	n	N	n/N()	x.y (95% CI)
^a^Age (weeks)							
2–12	n	N	n/N ()	n	N	n/N ()	x.y (95% CI)
13–40	n	N	n/N ()	n	N	n/N ()	x.y (95% CI)
41–104	n	N	n/N ()	n	N	n/N ()	x.y (95% CI)
105–260	n	N	n/N ()	n	N	n/N ()	x.y (95% CI)

^a^Age-stratified analyses in year 4

#### Timing of secondary endpoint assessments

The secondary endpoint of VT pneumococcal carriage prevalence among all ages in the population will be assessed in year 3 (tertiary endpoint) and year 4 (secondary endpoint) of the trial. Radiological pneumonia is measured during continuous population-based clinical surveillance and analysis will include all events in children enrolled to receive the intervention.

#### Timing of tertiary endpoint and disease endpoint assessment

The tertiary endpoint of VT carriage in infants aged 6–12 weeks who have not yet received their first dose of PCV will be assessed in year 4. Disease endpoints will be continuously measured in population-based clinical surveillance throughout the course of the trial. The disease endpoints will be assessed cumulatively during the 4 years of intervention and within each of the 4 years of intervention. The non-pneumococcal invasive bacterial disease endpoint is included as a control condition to detect potentially differential case ascertainment or access to care in the two groups. Supplementary Table 3 shows the results of disease endpoints in the trial.

## Statistical principles

### Confidence intervals and p-values

#### Level of statistical significance

The level of statistical significance for all non-inferiority hypotheses will be set at an α-level of 0.05 using two-sided significance tests [18, 19]. We specify a two-sided α-level of 0.05 as we wish to know the lower, as well as upper limit of the confidence interval around the effect estimates, as it is plausible that the alternative schedule may be superior to the standard schedule. For the superiority hypothesis concerning VT prevalence in children with clinical pneumonia in year 2, we will use a two-sided significance test with an α-level of 0.05.

#### Multiplicity

No adjustment will be made to the level of significance for multiple hypothesis tests. Hypothesis tests will be presented with a specific *p*-value for researchers to interpret within the context of all presented results and all the available evidence in the field [20].

#### Confidence intervals

Two-sided 95% confidence intervals will be used for all non-inferiority endpoints [18, 19] and the superiority analysis of VT prevalence in patients with clinical pneumonia endpoint in year 2. To account for the effect of clustering, 95% confidence intervals will be constructed using generalised estimating equations (GEE) with exchangeable correlation structure, based on individual-level data and a regression model using a log link function, binomial family, and robust standard errors. Confidence intervals for incidence rate ratio outcome measures will be calculated following specification of a cluster-level analysis using a negative binomial distribution. The negative binomial distribution allows an overdispersion parameter that accounts for clustering and repeated events, while cluster-level person-time will function as weights in the analysis. Confidence intervals for incidence rate ratios will be calculated by dividing and multiplying the point estimate by an error factor based on the *t*-distribution [21].

### Adherence and protocol deviations

#### Definition and assessment of adherence to the intervention

##### Vaccination status—per-protocol

Vaccination per-protocol (PP) will be defined as a child enrolled into the study at ≤273 days (≤40 weeks) of age and who received the allocated schedule of PCV doses during follow-up. PP administration of the standard schedule of three doses will require allocation to the standard schedule and administration of three doses of PCV at ≤152 days (≤22 weeks) of age. PP administration of the alternative schedule will require administration of two doses of PCV, the first dose ≤152 days (≤22 weeks) of age, and administration of the second dose ≥273 days (≥40 weeks) and ≤365 days (≤52 weeks) of age.

##### Vaccination status—intention-to-treat

Intention-to-treat (ITT) vaccination is defined as an enrolled child receiving the allocated schedule but with administration outside the PP criteria. ITT administration of the standard schedule will require administration of three or four doses of PCV with the final dose <252 days (<36 weeks) of age. ITT administration of the alternative schedule will be defined as administration of two doses of PCV with administration of the first dose ≤182 days (≤26 weeks) of age and administration of the second dose ≥336 days (≥48 weeks) of age.

##### Vaccination status—incomplete vaccination

Incomplete vaccination with the standard schedule will be defined as administration of only one dose of PCV, or administration of two doses of PCV with the second dose administered at ≤252 days (≤36 weeks) of age. Incomplete vaccination with the alternative schedule will be defined as the administration of only one dose of PCV, at any age. For individual-level ITT analyses, children with incomplete vaccination will be assigned to the group to which they were randomised.

##### Vaccination status—cross-over

Participants resident in a village allocated to the standard schedule will be defined as cross-over between groups if doses of PCV are received at an age when a dose will function as a booster, as is the intention of the alternative schedule. For infants allocated to the standard schedule, cross-over is defined as administration of (a) two doses of PCV with the second dose administered at ≥252 days (≥36 weeks) of age with an interval of >152 days (>21 weeks), or (b) three doses of PCV with the third dose administered at ≥252 days (≥36 weeks) of age with an interval of >152 days (>21 weeks) between the first and third doses, or (c) four doses of PCV with the fourth dose administered at ≥252 days (≥36 weeks) of age.

The definition of cross-over for infants resident in a village allocated to the alternative schedule is related to receiving three or more doses of PCV at an age when doses will not function as a booster, as is the intention of the standard schedule. For infants allocated to the alternative schedule, cross-over is defined as the administration of (a) three doses of PCV with the administration of the third dose <252 days (<36 weeks) of age, or (b) four doses with the administration of the fourth dose <252 days (<36 weeks) of age. Infants that migrate internally between clusters with alternate group allocation, before completing their PCV schedule, may validly change group allocation to the destination cluster. Internal migrations between clusters with alternate group allocation after completing their PCV schedule will be classified as cross-overs from the time of the migration.

##### Vaccination status—unvaccinated

Children who receive zero doses of PCV will be designated as unvaccinated.

##### Vaccination status—out-migration or death before age to complete vaccination

Vaccination status in individual-level analyses will be defined as out-migration or death before age to complete if the child migrates out at an age before which their PCV schedule could reasonably be completed. Infants resident in standard schedule villages who out-migrate or die before age ≤112 days (16 weeks) and receive zero doses of PCV will be classified as ‘standard schedule, out-migration/death, unvaccinated before age 16 weeks’ and ‘standard schedule, out-migration/death, incomplete before age 16 weeks’ if one or two doses are received. Infants resident in alternative schedule villages who out-migrate or die before age ≤294 days (≤42 weeks) and receive zero doses of PCV will be classified as ‘alternative schedule, out-migration/death, unvaccinated before age 42 weeks’ and ‘alternative schedule, out-migration/death, complete before age 42 weeks’ if one dose of PCV is received.

##### Assessment of individual-level adherence

Individual-level adherence will be assessed as (a) PP vaccination, (b) ITT vaccination, (c) incomplete vaccination, (d) cross-over, (e) unvaccinated, or (f) migration/death before completion. Individual-level adherence will be assessed cumulatively over the 4 years of intervention and reported as proportions of participants enrolled at EPI clinics who fall into these mutually exclusive categories.

##### Assessment of population-level adherence

Population-level adherence to the intervention, or the extent of exposure to the two schedules in the populations of the two groups, will be assessed according to eight mutually exclusive categories (Table 3). Population-level adherence will be assessed by group, on every day of the 4-year intervention period, with all resident children aged 0–260 weeks categorised on each day according to the eight categories. The age at which children are considered eligible refers to the earliest age at which children may have received doses of PCV, taking into account the predominant timing of EPI clinics once per month. That is, the first dose of PCV is scheduled to be given at 6 weeks of age, while the first opportunity to receive the first dose is when the monthly EPI clinic is held and an infant is aged 6 weeks or greater, which may be when the infant is 10 weeks of age. A dose of PCV administered before 6 weeks of age is considered ‘effective’ given the known immune response when PCV is administered in the neonatal period [22, 23].

**Table 3:** Population-level adherence to the intervention for resident children aged 0–260 weeks

	Alternative schedule	Standard schedule
Ineligible 0 doses	Age <10 weeks and 0 doses	Age <10 weeks and 0 doses
Eligible 0 doses	Age ≥10 weeks and 0 doses	Age ≥10 weeks and 0 doses
Eligible 1 dose	Age <44 weeks and 1 dose	Age <14 weeks and 1 dose
Eligible 2 doses	Age ≥36 weeks and 2 doses and PCV2 ≥36 weeks	Age <18 weeks and 2 doses
Eligible 3 doses	3 doses and age PCV3 ≥36 weeks	3 doses and age PCV3 <36 weeks
Eligible 4 doses	Enrolled 2 Sept 2019–7 Feb 2020 and 4 doses and age PCV4 ≥36 weeks	4 doses and age PCV4 <36 weeks
Incomplete	Age ≥36 weeks and 1 dose Age <36 weeks and 2 doses Age ≥36 weeks and 2 doses and age PCV2 <36 weeks	Age ≥14 weeks and 1 dose Age ≥18 weeks and 2 doses
Cross-over	3 doses and age PCV3 <36 weeks 4 doses and age PCV4 <36 weeks	3 doses and age PCV3 ≥36 weeks 4 doses and age PCV4 ≥36 weeks

#### Presentation of adherence to the intervention

##### Presentation of adherence among participants enrolled to receive interventions

Adherence among individual enrolled participants will be presented in tabular and graphical forms. A frequency table will show the proportions of all enrolled participants in each group who fall into the mutually exclusive categories of adherence described on p. 12 and 13. A histogram with stacked bars will represent the proportions of all enrolled participants in each group who fall into the categories.

##### Presentation of adherence among children in resident population

Adherence among resident children, or the extent of exposure to the two schedules in the populations resident in the villages allocated to the two groups, will be presented in graphical form. The proportions of resident children aged 0–260 weeks who fall into the mutually exclusive groups defined in Table 3 will be calculated on each day of the 4-year trial period and a histogram plotted with vertically stacked bars to a 100% total for every day over the 4-year period. In order to illustrate the increasing exposure of the population in the alternative schedule villages to the alternative schedule, the plot will demonstrate the increasing proportion over time of resident children who fall into the Table 3 adherence categories of ‘eligible 2 doses’, ‘eligible 3 doses’, and ‘eligible 4 doses’ over the 4-year trial period.

#### Definition of protocol deviations

Table 4 defines the protocol deviations in the trial. Enrolment of ineligible participants occurs when non-residents or infants greater than the age limit are enrolled. Incorrect administration of PCV doses occurs when infants enrolled in the alternative schedule group receive a second or third dose of PCV before 40 weeks of age, and in the standard schedule group if the third dose is administered after 40 weeks of age.

**Table 4:** Definition of protocol deviations

**Reason for protocol deviation**	
Enrolment of ineligible participant	Age >40 weeks on day of consent Non-resident on day of consent
Enrolment without consent	Enrolled without valid consent form
**Non-adherence to vaccine schedule**	
Incorrect dose given	
Standard schedule—received >3 doses	
Standard schedule—3rd dose age >40 weeks	
Alternative schedule—2nd dose age <40 weeks	
Alternative schedule—3rd dose age <40 weeks	
Ineligible and received alternative schedule	Non-resident—2nd dose age ≥36 weeks
Withdrawn and received alternative schedule	Withdrawn—2nd dose age ≥36 weeks

#### Which protocol deviations will be summarised

Protocol deviations will be summarised in Table 4.

### Analysis of populations and cohorts

#### Population-level analysis of populations and cohorts

##### Primary endpoint

Estimation of the impact of the schedules in the population incorporates both direct and indirect effects of the schedules in those aged <5 years in the geographic area. Measurement of the primary endpoint in year 4 of intervention allows time for the development of potentially differential impacts of the two schedules. The primary endpoint will be measured in all resident children aged 14–1826 days (2–260 weeks) presenting to health facilities in the study area with clinical pneumonia in year 4, assigning them to each group according to the group allocation of their village of residence, and regardless of whether the child was enrolled as an infant to receive the study intervention or number of PCV doses received (Table 1). Children eligible for selection for measurement of the primary endpoint will be (a) born in the HDSS, (b) resident in the HDSS in year 4, (c) aged 2–260 weeks on the day of presentation, and (d) present with clinical pneumonia at a health facility in the trial area. Neonates aged <14 days are excluded as pneumococcal carriage is low at this age and to avoid potential ascertainment bias due to clinical events in the early neonatal period that are unrelated to pneumococcal transmission. The primary endpoint is a measure of VT prevalence in children with clinical pneumonia and does not require follow-up. VT carriage prevalence in each trial group will be calculated with the denominator being number of children eligible for measurement.

**Fig. 1:**
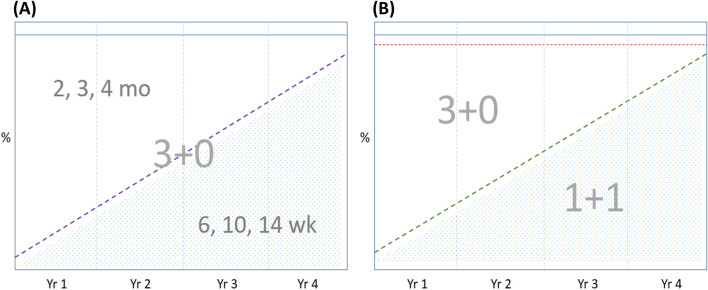
Study timeline of percentage vaccination coverage over time among all resident children aged 0–260 weeks in the **A** standard 3+0 and **B** alternative 1+1 schedule groups

PVS is a pragmatic trial under real-world conditions that aims to compare the impact of the two schedules in the population, taking into account direct and indirect effects. Figure 1 illustrates how coverage of the schedules will change over the 4 years of intervention, and by extension the timeline over which the impact of the schedules will develop. Figure 1A shows the projected coverage of the trial standard schedule (6, 10, 14 weeks) increasing over time (shaded area) in the allocated clusters. The projected coverage of the national standard schedule (2, 3, 4 months) decreases over time. A small proportion of children in the population never attend EPI clinics (~5%) and so maximum coverage is <100%. Figure 1B shows the projected coverage of the alternative schedule (6 weeks, 9 months) increasing over time (shaded area). Children in the population who never attend EPI clinics never receive PCV (~5%) and a proportion of children decline consent for the study and receive the standard schedule (~3%). Figure 1 shows that in year 4, coverage of the alternative schedule will still be increasing and not yet have reached maximal coverage. Figure 1B illustrates that in year 4, children represented by the shaded section will experience the direct and developing indirect effects of the 1+1 schedule, while those represented by the unshaded section will experience the direct effects of the 3+0 schedule and the developing indirect effects of the 1+1 schedule. Thus, in year 4, the majority of alternative schedule children aged 2–260 weeks will be experiencing the direct and indirect effect of the 1+1 schedule but a small proportion will be experiencing a mixture of effects of the standard and alternative schedules.

The primary endpoint will be analysed at the population-level including all resident children aged 2–260 weeks, born in the HDSS, who present with clinical pneumonia in year 4 assigning them to each group according to the group allocation of their village of residence regardless of whether the child was enrolled as an infant to receive the study intervention and regardless of PCV schedule or number of doses received (Table 1). Eligible children will be aged 14–1826 days on the day of presentation to the health facility during year 4. The first day of study intervention is defined as the day on which all eligible infants in the population had an opportunity to be enrolled. The first dose of study intervention was administered on 2 September 2019 and the first day on which all eligible infants in the population at the time had an opportunity to be enrolled was 7 October 2019. Thus, the first day of year 4 of intervention will be 7 October 2022. Year 4 surveillance will begin on 1 November 2022.

##### Other population-level endpoints

The population-level prevalence of VT pneumococcal carriage will be measured in years 3 and 4 using age-stratified random samples of all residents in the HDSS (Table 1). Age-stratified random samples of 60 participants will be selected in each cluster with a total sample size of 4080 in each year. Ten individuals in each randomly selected household will be randomly selected, 2:2:2:1:1:1:1 according to the age stratification (0–11 months, 12–23 months, 24–59 months, 5–9 years, 10–14 years, 15–44 years, and ≥45 years). The prevalence of VT carriage will be calculated for each cluster using the number of enrolled participants as the denominator.

The population-level prevalence of VT pneumococcal carriage in infants aged 6–12 weeks unimmunised with PCV will be measured in year 4 (Table 1). Eligible infants will be born to women resident in the HDSS, present to an EPI clinic, be 42–84 days of age, and not yet be immunised with a first dose of PCV. Sixty infants in each cluster will be randomly selected from eligible infants. The prevalence of VT carriage will be calculated for each cluster using the number of enrolled infants.

The incidence of radiological pneumonia will be calculated in each year of the study in population-level cohorts and over the 4-year period of intervention. Population-level cohorts will include all resident children 0–1826 days of age with follow-up starting on the day of birth or day of migration into the HDSS, or the follow-up start date, whichever is later. Follow-up ends at death, migration out of the HDSS, at 1826 days of age, or the end of the follow-up period, whichever is earlier. Follow-up observation time for children will be assigned according to the group allocation of their village of residence at the beginning of follow-up, regardless of later episodes of internal migration, whether the child was enrolled as an infant to receive the study intervention, and regardless of the PCV schedule or number of doses received. Repeated events in an individual will be included as the overdispersion parameter in the negative binomial model will account for the non-independence of these events. Follow-up observation time for children will be assigned according to the group allocation of their village of residence taking into account later episodes of internal migration, but regardless of whether the child was enrolled as an infant to receive the study intervention and regardless of the PCV schedule or number of doses received.

Analysis of VT IPD incidence will be calculated in each year of the study and over the 4-year period of intervention, at the population-level including resident children 0–1826 days of age with follow-up defined as for radiological pneumonia. Endpoints of all IPD and NVT IPD incidence (Table 1) will be analysed at the population-level using the same populations as defined for VT IPD incidence.

The clinical pneumonia incidence endpoint will include all resident children aged 0–1826 days. The incidence of clinical pneumonia will be calculated in each year and over the 4 years of intervention using the same populations as for radiological pneumonia.

Analysis of hypoxic pneumonia incidence will include all resident children aged 0–1826 days. The incidence of hypoxic pneumonia will be calculated in each year and the 4-year period of intervention using the same populations as for radiological pneumonia.

The analysis of incidence of clinical pneumonia associated with NP carriage of VT pneumococci will include resident children aged 14–1826 days. Incidence will be calculated in each year and over the 4 years of intervention using the same populations as for radiological pneumonia.

The incidence of hospitalisation will be calculated including all resident children 0–1826 days of age. Incidence will be calculated in each year and the 4 years of intervention using the same populations as defined for radiological pneumonia.

Analysis of mortality will include all resident children 0–1826 days of age. Incidence will be calculated in each year and over the 4 years of intervention using the same populations as defined for radiological pneumonia.

Analysis of the incidence of non-pneumococcal invasive bacterial disease and diarrhoea will include all resident children 0–1826 days of age. Incidence will be calculated in each year and over the 4-years of intervention in the same populations as defined for radiological pneumonia.

#### Individual-level cohorts

Individual-level ITT and PP cohorts will only include children enrolled as infants at EPI clinics to receive the study intervention. The individual-level ITT cohort will be used to calculate the prevalence of VT pneumococcal carriage in resident study participants presenting with clinical pneumonia in year 4. The individual-level ITT cohort will include all resident participants enrolled to receive study interventions who present with clinical pneumonia, assigning them to the study groups according to the allocation of their village of residence at enrolment, regardless of age at enrolment, the schedule, or number of doses received. The denominator in prevalence calculations will be the number of eligible children with clinical pneumonia. Repeated events in one individual will be included with the related non-independence of events accounted for in the cluster-level effect included in the GEE model.

The individual-level PP cohort will be used to calculate the prevalence of VT pneumococcal carriage in study participants presenting with clinical pneumonia in year 4. The individual-level PP cohort will include fully vaccinated resident participants (see the ‘Definition and assessment of adherence to the intervention’ section), enrolled at ≤273 days of age who present with clinical pneumonia, assigning them to the study groups according to the village of residence at enrolment. The denominator in prevalence calculations will be the number of eligible children with clinical pneumonia in the individual-level PP cohort. Repeated events will be included.

Incidence endpoints will be analysed using the individual-level ITT cohort including all participants enrolled to receive the intervention, assigning them to the study groups according to the allocation of their village of residence at enrolment, regardless of age at enrolment, the schedule, or number of doses received. Event counting starts on the date of enrolment. Follow-up ends either at death, outmigration, or the end of the follow-up period, whichever is earlier.

Incidence endpoints will also be analysed using the individual-level PP cohort including participants enrolled to receive the intervention, enrolled at ≤273 days of age, are fully vaccinated (see the ‘Definition and assessment of adherence to the intervention’ section), assigning them to a study group according to the village of residence at enrolment. Event counting starts 14 days after the administration of the first dose of PCV. Follow-up ends either at death, migration out of the HDSS, or the end of the follow-up period, whichever is earlier.

Individual-level cohort analyses using ITT and PP cohorts will calculate the incidence of radiological pneumonia and VT IPD. VT IPD is the most specific endpoint able to detect potentially differential efficacy of the two schedules; however, small numbers of events will limit the power to detect differences. Radiological pneumonia is an endpoint of unknown, but likely only modest specificity, and may be able to detect differences between the schedules. Although numbers of events are significant, the number is limited and may not have sufficient power to detect differences. Individual-level analyses using ITT and PP cohorts will also calculate the incidence of clinical pneumonia, hypoxic pneumonia, clinical pneumonia associated with NP carriage of VT, NVT, any pneumococci, hospitalisation, mortality, non-pneumococcal bacteraemia, and diarrhoea.

## Trial population

### Screening

The trial is being conducted in the BHDSS and FWHDSS in rural Gambia. All resident infants are eligible for enrolment at EPI clinics to receive the trial interventions. All resident children aged 0–260 weeks are under surveillance for the primary endpoint and clinical disease endpoints. The year 3 and 4 population-level carriage surveys will include cluster-wise random samples of resident individuals. The year 4 carriage survey in infants aged 6–12 weeks yet to be immunised with PCV will include 60 randomly selected infants per cluster presenting to EPI clinics at the mid-point of year 4. The trial samples are representative of the population.

We will compare the numbers and characteristics of resident children aged 0–260 weeks, born in the trial area, who (a) never present to EPI clinics, (b) present only once to an EPI clinic, and (c) present on multiple occasions to EPI clinics. Characteristics will include sex, mother’s age at the child’s birth, number of household members, number of household children aged <15 years, ethnicity, and mortality.

### Eligibility

Participants must be resident in the study area with an HDSS 14-digit individual ID number. Exclusion criteria for enrolment at an EPI clinic are as follows: intention to out-migrate before 4 months of age, completed PCV schedule, contraindication to PCV, or age >274 days (or age >182 days in the standard schedule group from 22 August 2019 to 7 February 2020).

### Recruitment

**Fig. 2:**
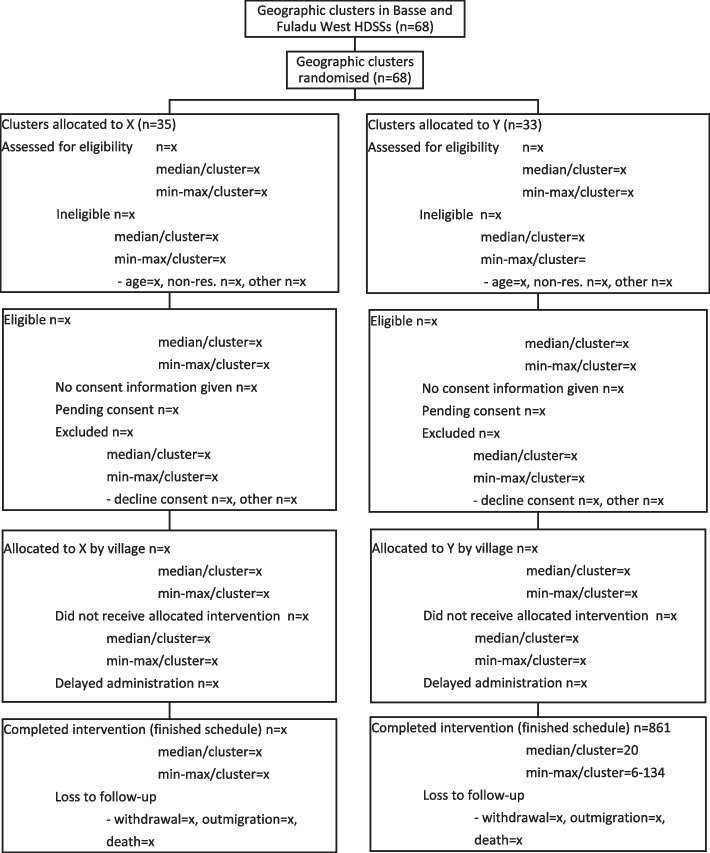
CONSORT diagram of infant enrolment at EPI clinics to receive the intervention

Information to include in the CONSORT flow diagram of enrolment is illustrated in Fig. 2.

### Withdrawal/follow-up

Participants may withdraw at any time from receipt of the alternative schedule intervention but may not withdraw from surveillance for measurement of endpoints. We will continue to record vaccines received by withdrawn participants. Children who migrate out of the trial area before 260 weeks of age will be lost to follow-up. We will present the number of withdrawn and out-migrated participants in the CONSORT flow diagram (Fig. 2).

### Baseline characteristics

#### Descriptive summary of enrolment of infants at EPI clinics

A histogram will illustrate the number of infants screened for enrolment at EPI clinics each month, the number eligible, and the number enrolled, by group (supplementary figure 1, does not show group-wise enrolment). A line graph will illustrate the cumulative number of infants screened, eligible, and enrolled at EPI clinics, by group (supplementary figure 2, does not show group-wise enrolment). Descriptive analyses will tabulate the total number of infants enrolled at EPI clinics and allocated to each group, by year of enrolment, and by cluster. The characteristics of all infants enrolled at EPI clinics will be summarised at the individual-level, by group (supplementary table 4) with further disaggregation by study year (supplementary table 5). Infant characteristics will also be summarised at the cluster-level (supplementary table 6) [3].

A sub-analysis of individual-level characteristics of infants will be restricted to the first 5 ½ months of the study, between 22 August 2019 and 7 February 2020. Per-protocol enrolment between 22 August 2019 and 7 February 2020 specified enrolment of infants up to 9 months of age in alternative schedule villages. In standard schedule villages, enrolment criteria during this period included infants who had not yet received the 3rd dose of PCV or were less than 6 months of age (see the ‘Eligibility’ section). Thus, we will summarise the baseline characteristics of infants aged 6–9 months, resident in standard schedule villages between 22 August 2019 and 7 February 2020 and not enrolled along with enrolled infants aged 6–9 months and resident in alternative schedule villages during the period of interest.

#### Descriptive analysis of intervention delivery

Descriptive analysis of the delivery to the intervention to individual infants enrolled at EPI clinics and at the level of the population resident in the geographic clusters has been presented in the ‘Presentation of adherence to the intervention’ section.

#### Descriptive analysis of the surveillance population

Descriptive analysis of the surveillance population will summarise by group, the number of residents aged 0–260 weeks at the mid-point of each year, along with the number of births, deaths, out-migrations, in-migrations, internal migrations (with or without cross-over) in each year of surveillance, and the cumulative numbers of events over the 4 years of surveillance. Person-years under observation will also be presented and calculated with the end of an individual observation period given by the date of death, out-migration, or end of trial follow-up. An individual may contribute multiple periods of observation associated with potential out-migration and later return to the trial area.

### Descriptive analysis of endpoint surveillance at health facilities

We will report the number of resident patients aged 2–260 weeks presenting to health facilities, by cluster, and the number with clinical pneumonia. Descriptive analysis of the primary endpoint surveillance at health facilities and determination of the primary endpoint will be summarised in supplementary figures 3 and 4. Descriptive analysis of surveillance for clinical endpoints at health facilities will be summarised in supplementary tables 7 and 8, including summary statistics at the individual-level and cluster-level [3]. Baseline characteristics in clinical surveillance will be reported in each year and cumulatively, including number under surveillance; age and sex of children presenting at health facilities as well as children with clinical pneumonia; numbers aged 0–28 days, 4–52 weeks, and 52–260 weeks; nutritional status of admitted children; rapid malaria test results; and haemoglobin values. The incidence of blood cultures and chest radiographs and the proportion of those investigations that are positive will indicate potential bias in case ascertainment and access to care. The numbers and proportions of patients with clinical pneumonia who are hospitalised and who have chest radiographs will also indicate potential bias in case ascertainment and access to care.

### Descriptive analysis of enrolment in the year 3 and 4 surveys of pneumococcal carriage

Descriptive analysis of enrolment in the population-level community surveys of pneumococcal carriage in years 3 and 4 will report the number enrolled by trial group, the median number per cluster, and the number and proportion consenting to participate. Baseline characteristics will be reported by group overall and in age strata: age, sex, urban residence, cough or rhinorrhea (participant and household members), antibiotic use (in preceding 14 days), smoker in household, number of people sharing a bed, number of children aged <5 in household, household cooking fuel, participant presence in cooking area, hand washing, and masking.

### Descriptive analysis of enrolment in the year 4 survey of pneumococcal carriage in infants aged 6–12 weeks yet to be immunised with PCV

Descriptive analysis will present the number of participants enrolled, by trial group, the median number per cluster, and the number and proportion consenting to participate. Baseline characteristics will include age, sex, urban residence, cough or rhinorrhoea (participant and household members), antibiotic use (in preceding 14 days), smoker in household, number of people sharing a bed, number of children aged <5 in household, household cooking fuel, presence of the infant in the cooking area, hand washing, and masking of carers.

## Analysis

### Outcome definitions

#### NP carriage of VT pneumococci in children with clinical pneumonia

We have chosen NP carriage of VT pneumococci in children with clinical pneumonia as the primary endpoint because the greatest public health burden of pneumococcal infection is due to pneumonia, it is a correlate of pneumococcal transmission in children in the population, there is a correlation with pneumonia aetiology, and this patient sub-group is relatively easy to access for investigation. Clinical pneumonia is defined as cough or difficulty breathing for <14 days accompanied by a raised respiratory rate for age or lower chest wall in-drawing or O_2_ saturation <93% in children treated as outpatients, and among hospitalised children accompanied by one or more of the additional signs and symptoms: nasal flaring, grunting, stridor, gasping, head nodding, history of convulsion, inability to sit or feed, vomiting everything, lethargy, impaired consciousness or dullness to percussion, bronchial breathing, or coarse crackles detected by a clinician. Vaccine-type pneumococcal carriage is defined as detection of one or more of the serotypes included in PCV13: 1, 3, 4, 5, 6A, 6B, 7F, 9V, 14, 18C, 19A, 19F, and 23F. Cross-reactive serotype 6C is defined as a VT [23–25]. Non-typeable serotypes will be excluded. This definition will be applied at the population-level in children aged 14–1826 days and 14–1638 days, as well as at the individual-level in PP and ITT cohorts.

#### NP carriage of VT pneumococci

Vaccine-type pneumococcal carriage is defined in the ‘NP carriage of VT pneumococci in children with clinical pneumonia’ section. Non-typeable serotypes will be excluded. This definition will be applied to all participants in the year 3 (tertiary endpoint) and year 4 (secondary endpoint) cross-sectional surveys of pneumococcal carriage.

#### Radiological pneumonia

Radiological pneumonia is defined according to the WHO standard for radiological pneumonia in children [26]. In order to determine radiological pneumonia, the quality of a radiograph must be ‘adequate’ or ‘suboptimal’ for both independent readers, or for one independent reader and the discordant film reader. Radiographs will be excluded if ‘uninterpretable’ by both independent readers or by one independent reader and the discordant film reader. Readers classify radiograph quality as follows:

1. *Uninterpretable*: not interpretable in terms of the presence or absence of ‘end-point consolidation’
2. *Suboptimal*: allows interpretation of end-point consolidation but not of other infiltrates
3. *Adequate*: allows interpretation of end-point consolidation as well as other infiltrates

Radiograph readers classify the radiograph findings as follows:

A) *End-point consolidation or pleural effusion*: end-point consolidation (dense opacity, may be fluffy consolidation of a portion or whole of a lobe, often containing air bronchograms) or pleural effusion (fluid in the pleural space, often at the costo-phrenic angle or as a layer of fluid adjacent to the lateral chest wall, spatially associated with a parenchymal infiltrate or if effusion obliterates the hemithorax enough to obscure an opacity)
B) *Other consolidation/infiltrate*: other (non-end-point) infiltrates of linear or patchy densities in a lacy pattern involving both lungs featuring peribronchial thickening or patchy atelectasis, or small areas of atelectasis difficult to differentiate from consolidation, in the absence of a pleural effusion
C) *No consolidation/infiltrate/effusion:* absence of end point consolidation, other infiltrate or pleural effusion

If multiple radiographs are taken during an episode of illness, the worst radiographic appearance in the 3 days following the date of admission will be accepted at final. Multiple episodes for one child will be considered separate events if the first and subsequent consultations are at least 30 days apart. This definition will be applied at the population-level (Table 1) and in individual-level PP and ITT cohorts.

#### NP carriage of VT, NVT, and all pneumococci in PCV unimmunised infants aged 6–12 weeks

The definition for VT carriage is as for the ‘NP carriage of VT pneumococci in children with clinical pneumonia’ section. NVT carriage is defined as the detection of one or more serotypes excluding all those in PCV13 and cross-reactive serotype 6C. Non-typeable serotypes will be excluded. This definition will be applied to infants aged 6–12 weeks unimmunised with PCV and enrolled in the year 4 survey.

#### NP carriage of NVT and all pneumococci

The NVT definition is as for outcome ‘NP carriage of VT, NVT, and all pneumococci in PCV unimmunised infants aged 6–12 weeks’ section and will be applied to all participants in the year 3 and year 4 cross-sectional surveys of pneumococcal carriage.

#### Clinical pneumonia

The case definition for clinical pneumonia is the same as for the primary endpoint and will be applied at the population-level and in individual-level PP and ITT cohorts.

#### Hypoxic clinical pneumonia

The clinical pneumonia definition is the same as for the primary endpoint and will be associated with O_2_ saturation <91% and be applied at the population-level and in individual-level PP and ITT cohorts.

#### Clinical pneumonia associated with NP carriage of VT, NVT, and all pneumococci

The case definition for clinical pneumonia with carriage of VT pneumococci is the same as for the primary endpoint. NVT carriage is defined as in the ‘NP carriage of VT, NVT, and all pneumococci in PCV unimmunised infants aged 6–12 weeks’ section. These definitions will be analysed as incidence and prevalence endpoint and applied at the population-level and in individual-level PP and ITT cohorts.

#### VT, NVT, and all IPD

Vaccine-type IPD will be defined as isolation of *Streptococcus pneumoniae* from a sterile site from a patient with a surveillance diagnosis of clinical pneumonia, sepsis, meningitis, focal infection, or other medical problem. Vaccine-types will be defined as in the ‘NP carriage of VT pneumococci in children with clinical pneumonia’ section and non-vaccine types as in the ‘NP carriage of VT, NVT, and all pneumococci in PCV unimmunised infants aged 6–12 weeks’ section. Non-typeable serotypes will be excluded. Episodes will be considered separate events if 30 days apart, or a different serotype is isolated at each episode. Cases of pneumococcal disease in which two different serotypes are isolated will be classified as one case, but two different episodes if the two different serotypes belong to different vaccine-associated categories. These case definitions will be applied at the population-level and in individual-level PP and ITT cohorts and analysed as incidence endpoints.

#### Hospitalisation

Hospitalisation is defined as a child admitted overnight to one of the 11 health facilities in the study area, irrespective of diagnosis. This case definition will be applied at the population-level and in individual-level PP and ITT cohorts.

#### Mortality

Death of a child registered in the study electronic medical record at one of 11 health facilities in the study area or a death registered at a 4-monthly household enumeration in the HDSS. This case definition will be applied at the population-level and in individual-level PP and ITT cohorts.

#### Non-pneumococcal invasive bacterial disease

Non-pneumococcal invasive bacterial disease will be defined as isolation of any bacterial pathogen, other than *S. pneumoniae*, from a sterile site. Contaminants will be excluded. Episodes will be considered separate events if 30 days apart, or a different bacteria is isolated at each episode. Case in which two different pathogens are isolated will be classified as one case. This definition will be applied at the population-level and in individual-level PP and ITT cohorts.

#### Diarrhoeal disease

This will be defined as a patient complaint of diarrhoea, being three or more loose stool in one day.

### Case ascertainment

The details of case ascertainment are found in the supplementary material and published protocol [5].

### Unit of inference

The unit of inference is the individual child resident in the population. Therefore, analyses will be conducted at the level of the individual.

### Analysis methods

The framework for the analyses shown in Table 1 is expanded in detail in supplementary table 9, specifying the populations and cohorts, observations and follow-up, endpoints, and effect measures.

#### Population-level primary endpoint

The primary analysis will be assessment of the impact of the two schedules on VT carriage prevalence in resident children aged 2–260 weeks with clinical pneumonia. Interpretation will relate to whether the prevalence of VT carriage in a child who lives in a geographic area allocated to the alternative schedule is not greater than the non-inferiority threshold when compared to a child who lives in a geographic area allocated to the standard schedule.

The primary endpoint will be calculated as the proportion (prevalence) of children with clinical pneumonia who have NP carriage of VT pneumococci. The contrast between the two groups will be presented as a prevalence ratio, with ratios expressed as the prevalence in the alternative compared to standard schedule group. To account for cluster-level non-independence, the primary analysis will use generalised estimating equations (GEE) with a log link, binomial family, and exchangeable correlation structure. The primary analysis GEE model will include the stratifying covariates used in the randomisation of clusters: (i) cluster location in BHDSS or FWHDSS, (ii) cluster high or low baseline incidence of clinical pneumonia. Analysis will not include the other variables used to restrict the randomisation lists to ensure balance in the other baseline characteristics (see the ‘Randomisation’ section) [27]. Patients and clusters will be analysed according to their random allocation.

If the ratio of VT prevalence in the two groups is given by ρ = π_1_ : π_0_, where *ρ* is the true ratio between the prevalence in the alternative (π_1_) and standard (π_0_) schedule groups, the null hypothesis is H_0_ : *ρ* > 1.38, and the alternative hypothesis is H_1_ : *ρ* ≤ 1.38. The null hypothesis represents inferiority of the alternative schedule [13]. Non-inferiority will be established if the upper limit of the confidence interval around the prevalence ratio is ≤1.38.

The risk of measuring the endpoint in resident children aged 2–260 weeks with clinical pneumonia is dilution of potential differences between the two groups as a small proportion of children in their 4th year of life living in alternative schedule villages in year 4 will not have received the trial intervention and will experience the indirect effects of the alternative schedule but the direct effects of the standard schedule, introducing potential bias towards the null. This potential bias should be quite limited as the difference in direct effects of the schedules should be negligible in the 4th year of life.

#### Population-level VT prevalence in the community

The endpoint will be calculated as the proportion (prevalence) of VT pneumococcal carriage in all survey participants, by group, in the year 4 survey and also in the year 3 survey. The contrast between the two groups will be presented as a prevalence ratio (and 95% CI), with ratios expressed as the prevalence in the alternative compared to standard schedule group. To account for cluster-level non-independence, the analysis will use GEEs with a log link, binomial family and exchangeable correlation structure. The primary analysis GEE model will include the age-stratification in the survey design and the stratifying covariates used in the randomisation of clusters: (i) cluster location in BHDSS or FWHDSS and (ii) cluster high or low baseline incidence of clinical pneumonia. The clusters will be analysed according to their random allocation.

If the ratio of VT prevalence in the two groups is given by *ρ* = π_1_ : π_0_, where *ρ* is the true ratio between the prevalence in the alternative (π_1_) and standard (π_0_) schedule groups, the null hypothesis is H_0_ : *ρ* > 1.38, and the alternative hypothesis is H_1_ : *ρ* ≤ 1.38. The null hypothesis represents inferiority of the alternative schedule [13]. Non-inferiority will be established if the upper limit of the confidence interval around the prevalence ratio is ≤1.38.

#### Population-level VT prevalence in infants aged 6–12 weeks unimmunised with PCV

The endpoint will be calculated and compared in the same way as for VT prevalence in the community. The GEE model will include the stratification covariates and age (in days). The clusters will be analysed according to their random allocation. Non-inferiority will be established in the same way as for VT prevalence in the community. This endpoint will be compared using survey data from year 4.

#### Population-level incidence endpoints

Incidence endpoints include VT IPD, radiological pneumonia, clinical pneumonia, clinical pneumonia with VT carriage, hypoxic pneumonia, hospitalisation, mortality, non-pneumococcal invasive bacterial disease, and diarrhoeal disease in children aged 0–260 weeks. We will also measure the incidence of clinical pneumonia with VT carriage in children aged 2–260 weeks. The relative impact of the alternative compared to standard schedule at the population-level will be calculated using the following formula:

$$\textrm{Schedule}\ \textrm{impact}=1-\frac{\textrm{incidence}\ \textrm{in}\ \textrm{alternative}\ \textrm{schedule}\ \textrm{clusters}}{\textrm{incidence}\ \textrm{in}\ \textrm{standard}\ \textrm{schedule}\ \textrm{clusters}}\times 100$$

Incidence equals the number of cases divided by person-years at risk. We will assume that the number of cases follows a negative binomial distribution. We will calculate person-years at risk for each child in the population under surveillance from birth (or age 14 days for clinical pneumonia with pneumococcal carriage), migration into the trial area, or the start of trial observation, whichever is later until the end of trial observation, age 260 weeks, or death, whichever is earlier. We will include recurrent events given their importance as events of public health interest and use the negative binomial distribution overdispersion parameter to account for non-independence of events at the cluster level.

The contrast between the two groups will be presented as incidence rate ratios (with 95% CIs). Cluster-level summaries of incidence will be calculated using a negative binomial model including the stratification covariates used in randomisation, while the inclusion of person-time at risk will provide appropriate weights for each cluster. Confidence intervals for incidence rate ratios will be calculated by dividing and multiplying the point estimate by and error factor based on the t-distribution [21]. The clusters will be analysed according to their random allocation. A non-inferiority margin is not specified for these outcomes as the trial is not powered for these endpoints. In this respect, analysis of these disease incidence endpoints will not test any hypothesis but provide information to support the interpretation of trial results.

#### Individual-level cohort incidence endpoints

Incidence endpoints of VT IPD, radiological pneumonia, clinical pneumonia, clinical pneumonia with VT carriage, hypoxic pneumonia, hospitalisation, mortality, non-pneumococcal invasive bacterial disease, and diarrhoeal disease will be included in individual-level analyses. Analyses will be conducted using ITT and PP cohorts including participants enrolled to receive the intervention and participants who received the intervention respectively. Calculation of person-time at risk and the method of analysis will be the same as in the ‘Population-level incidence endpoints’ section.

#### Individual-level cohort VT prevalence

The endpoint of VT carriage prevalence in children with clinical pneumonia will be analysed using individual-level ITT and PP cohorts, including participants enrolled to receive the intervention and participants who received the intervention respectively. Calculation of prevalence ratios will use the same method of analysis as for the primary endpoint.

#### Population-level tertiary endpoints

Tertiary endpoints include the incidence of VT, NVT, all IPD, and non-pneumococcal invasive bacterial disease which will be analysed using the same methods as in the ‘Population-level incidence endpoints’ section. The endpoint of NVT prevalence in children with clinical pneumonia will be analysed at the population level using the same methods as for the primary endpoint.

#### Presentation of results

The results of non-inferiority analyses will be summarised in a figure showing the point estimates and confidence intervals for multiple endpoints in relation to vertical reference lines indicating the null effect and the non-inferiority margin. The figure will only include endpoints with sufficient statistical power to test the non-inferiority hypothesis.

#### Adjustment for covariates

As noted earlier, the stratifying covariates used in randomisation: location in BHDSS or FWHDSS and high-low cluster baseline incidence will be included in all statistical models. The other variables used to generate restricted randomisation lists to ensure balance in baseline characteristics will not be included in analyses [27]. Age will be included in models to estimate schedule impact on community-level VT carriage (via age stratification) and VT carriage in young infants (age in days). If differential case ascertainment or access to care is evident, we will consider further adjusted analyses.

#### Effect modification

We have a priori interest to examine the following potential effect modifiers for the primary endpoint: (a) age (2–12 weeks, 13 weeks to 9 months, 10–23 months, and 24–59 months) and (b) inpatient/outpatient status. The number of cases of each endpoint in these subgroups may not be sufficient to assess non-inferiority. We will examine the strength of evidence for differences between subgroups using interaction tests.

#### Assumptions to be checked for statistical methods

For regression models using GEE, we assume that observations within the same cluster may be correlated. We will use the common assumption in cluster-randomised trials that the correlation matrix is exchangeable, meaning that observations on individuals in different clusters are uncorrelated and that observations on individuals in the same cluster all have the same correlation coefficient. This assumption is usually correct for individuals living in the same community. The GEE method does not require a distributional assumption for cluster-level values.

Inference based on analyses of incidence using cluster-level summaries assumes a normal distribution of the observed cluster summaries [21]. We will inspect the distribution of the observed cluster summaries for skewness. If the cluster summaries in the incidence analyses are positively skewed, we will consider applying a logarithmic transformation to the incidence rates [21].

#### Alternative methods if distributional assumptions do not hold

If GEE regression analyses using the log link function for binary data do not converge, we will implement GEE regression analyses using a logit link functions. Prevalence ratios will be obtained following marginal standardisation and confidence intervals using the delta method.

#### Sensitivity analyses

No sensitivity analyses are planned.

#### Sub-group analyses

Analysis of the primary endpoint will be conducted in the subgroups specified in the ‘effect modification section’. We will also examine the relative schedule impact on VT carriage in the community, by survey age-strata, i.e. 0–11 months, 12–23 months, 2–4 years, 5–9 years, 10–14 years, 15–44 years, and ≥45 years.

### Missing data

Missing data will be reported in tables and figures. Specific missing data will include NP pneumococcal carriage and serotype for resident children with clinical pneumonia, NP carriage in the community surveys, blood culture and chest X-ray results for hospitalised patients with suspected pneumonia, sepsis, or meningitis, and group allocation. We do not expect missingness for these variables to be greater than 2–3%. If the percentage of missing data is greater than 10% for any of the specified variables, we will consider methods to impute missing values and sensitivity analyses with imputed data.

### Additional analyses

Analysis of the primary endpoint using data from year 2 of intervention (1 Nov 2020–31 Oct 2021) will be conducted with a superiority hypothesis, aiming to detect potential early effects of the interventions. The statistical model will estimate the prevalence difference (and 95% CI). Interpretation will be based on the a priori statement that the smallest significant difference is prevalence ratio of 1.47.

Analysis will include group-wise calculation of the intra-cluster correlation coefficients for VT pneumococcal carriage in children with clinical pneumonia, population-based carriage survey in year 4, and the survey of carriage in young infants in year 4.

We will explore potential differential risk of events in the alternative schedule group before and after administration of the booster dose. We will calculate incidence rate ratios for radiological pneumonia and hypoxic pneumonia among those who received PCV per-protocol (see the ‘Definition and assessment of adherence to the intervention’ section). We will count events from 14 days after administration of the first dose of PCV, before administration of the second dose (alternative schedule group), and ≤288 days of age (9 months and 2 weeks). We will also count events from 7 days after administration of the second dose of PCV (alternative schedule group) and >288 days of age. These analyses will isolate the period of potential inferiority before the booster dose in the alternative schedule group.

*Safety*

PCV13 is a licensed product with an excellent safety profile. Safety at the individual level is best evaluated by comparison of infants enrolled at EPI clinics to receive the trial intervention schedules. In these safety analyses, the effects of the two schedules will not be diluted by the inclusion of children who may not have been enrolled to receive the interventions, as is done in population-level analyses. Thus, any differences between the two schedules will be more likely detected in these individual-level analyses. Individual-level ITT and PP safety analyses will include the endpoints of VT IPD, radiological pneumonia, and mortality incidence (see the ‘Individual-level cohort incidence endpoints’ section).

Serious adverse events (SAE), which will primarily be events of hospitalisation and deaths at home detected by the HDSS, will be reported in each group at the population-level (residents aged 0–260 weeks) and individual-level in infants enrolled to receive the interventions. Events of radiological pneumonia and VT IPD will be deemed SAEs of special interest, and reported in each group at the individual-level in enrolled infants.

Unsolicited reactogenicity events within 7 days of a dose of PCV recorded at EPI clinics will be reported in each group [5]. Severe adverse reactions to doses of PCV will be reported for each group, specifically episodes of anaphylaxis and severe local reactions. The probability of suspected unexpected severe adverse reactions (SUSAR) to doses of PCV will be reported based on the probability of any SAE in the 3 days following administration of dose two (age 10 weeks) or dose three (age 14 weeks) of PCV in the standard schedule group compared to 3-day period following these age points in the alternative schedule group. Similarly, we will compare the probability of an SAE in the 3 days following administration of the booster dose at age 9 months in the alternative schedule group compared to the 3-day period following this age point in the standard schedule group. These probabilities will be compared to indicate signals of potential SUSARs related to PCV.

### Statistical software

STATA version 17 (College Station, TX, USA) and R software will be used for analyses.

### References

#### Non-standard statistical methods

Not applicable.

#### Data management plan

The data management plan is available upon request.

#### Trial Master File

The Trial Master File is located at the MRCG at LSHTM Basse Field Station. Documents from the file are available upon request.

#### Standard operating procedures and study-specific procedures

These documents are available upon request.

## Supplementary Information

**Additional file 1: Supplementary Table 1.** Clinical definitions for suspected pneumonia, septicaemia, and meningitis. **Supplementary Table 2.** Guideline for investigation of patients. **Supplementary Table 3.** Disease endpoints in children aged 0 – 59 months. **Supplementary Table 4.** Individual-level baseline characteristics of all infants enrolled at EPI clinics. **Supplementary Table 5.** Individual-level baseline characteristics of infants enrolled at EPI clinics, by year. **Supplementary Table 6.** Cluster-level baseline characteristics of infants enrolled at EPI clinics. **Supplementary Figure 1.** Monthly number of infants screened, eligible, and enrolled at EPI clinics. **Supplementary Figure 2.** Cumulative number of infants screened, eligible, and enrolled at EPI clinics. **Supplementary Figure 3.** Flowchart of patient presentation and eligibility for measurement of the primary endpoint at health facilities. **Supplementary Figure 4.** Flowchart of patient investigation and results of primary endpoint surveillance at health facilities. **Supplementary Table 7.** Baseline information on clinical endpoint surveillance at health facilities, at the individual-level. **Supplementary Table 8.** Baseline information on endpoint surveillance at health facilities, at the cluster level.

## Data Availability

The Principle Investigator and Trial Statistician will have access to the final trial dataset. Datasets will be available from the Principle Investigator, via application to the MRCG Scientific Coordinating Committee and GG/MRCG JEC.

## Abbreviations

BHDSS: Basse Health and Demographic Surveillance System
DMC: Data monitoring committee
EPI: Expanded programme on immunisation
FWHDSS: Fuladu West Health and Demographic Surveillance System
GG/MRCG JEC: Gambia Government/Medical Research Council Unit The Gambia Joint Ethics Committee
GEE: Generalised estimating equations
ICC: Intra-cluster correlation coefficient
ITT: Intention-to-treat
IPD: Invasive pneumococcal disease
LSHTM: London School of Hygiene & Tropical Medicine
MRCG: Medical Research Council Unit The Gambia
NP: Nasopharyngeal
NVT: Non-vaccine type
PCV: Pneumococcal conjugate vaccine
PP: Per-protocol
PVS: Pneumococcal vaccine schedules trial
Spn: *Streptococcus pneumoniae*
SAP: Statistical analysis plan
TSC: Trial steering committee
VT: Vaccine-type
