# Supplementary material for: Pneumococcal vaccine schedules (PVS) study: a cluster-randomised, non-inferiority trial of an alternative versus standard schedule for pneumococcal conjugate vaccination—statistical analysis plan
Source: Trials. 2022 Dec 28;23:1058. doi: 10.1186/s13063-022-06900-x (PMC9798555; doi:10.1186/s13063-022-06900-x)
Supplement: Supplementary file 1 — Additional file 1: Supplementary Table 1. Clinical definitions for suspected pneumonia, septicaemia, and meningitis. Supplementary Table 2. Guideline for investigation of patients. Supplementary Table 3. Disease endpoints in children aged 0 – 59 months. Supplementary Table 4. Individual-level baseline characteristics of all infants enrolled at EPI clinics. Supplementary Table 5. Individual-level baseline characteristics of infants enrolled at EPI clinics, by year. Supplementary Table 6. Cluster-level baseline characteristics of infants enrolled at EPI clinics. Supplementary Figure 1. Monthly number of infants screened, eligible, and enrolled at EPI clinics. Supplementary Figure 2. Cumulative number of infants screened, eligible, and enrolled at EPI clinics. Supplementary Figure 3. Flowchart of patient presentation and eligibility for measurement of the primary endpoint at health facilities. Supplementary Figure 4. Flowchart of patient investigation and results of primary endpoint surveillance at health facilities. Supplementary Table 7. Baseline information on clinical endpoint surveillance at health facilities, at the individual-level. Supplementary Table 8. Baseline information on endpoint surveillance at health facilities, at the cluster level. [file 13063_2022_6900_MOESM1_ESM.docx]

**Pneumococcal Vaccine Schedules study: a cluster-randomised, non-inferiority trial of an alternative versus standard schedule for pneumococcal conjugate vaccination**

## Statistical Analysis Plan

**Supplementary material**

**Case ascertainment**

**Clinical surveillance**

Patients aged <5 years are eligible for enrolment to measure endpoints if they are resident in the trial area and present as an outpatient, or are admitted to one of the health facilities in the trial area. Trial nursing staff use an electronic medical record to record demographic and clinical information for all unwell children presenting to each health facility. All resident patients aged <5 years undergo clinical pneumonia screening. The potential for ascertainment bias for the primary endpoint is minimized by measures to conceal group allocation from clinical assessors. Whenever possible, the staff who registers children’s attendance will conceal the infant welfare card (and residential location) from a second staff who performs the clinical pneumonia screening. The patient is classified and recorded as clinical pneumonia (which indicates collection of a NP specimen) if there is cough or difficulty breathing for <14 days and either the respiratory rate is raised for age, there is lower chest wall indrawing or oxygen saturation is <93%. After clinical pneumonia screening the second staff has access to the infant welfare care and the patient undergoes a full nursing evaluation. Study staff register and screen all unwell children presenting to the 11 health facilities from Monday to Saturday 0800-2000. Children presenting overnight between 2000 and 0800 or on Sunday are evaluated by Ministry of Health (MoH) staff. Children admitted overnight or on Sunday are evaluated and investigated by study staff the following morning or on Monday morning respectively. All study data are recorded in real-time in an electronic medical record (EMR) or on standardized paper forms if the EMR is unavailable.

Standardised criteria (supplementary table 1) are used to classify patients according to a surveillance diagnosis of suspected pneumonia, septicaemia, or meningitis or other diagnoses consistent with the scheme of Integrated Management Childhood Illness. According to the standardised diagnosis patients are investigated as per the guideline in supplementary table 2. Radiographs are performed in Basse, Bansang, Fatoto and Jakhaly. Patients are transported for X-ray at the closest location and then returned for ongoing care at the original health facility.

All children admitted with an acute medical problem have a blood culture taken, a plasma aliquot stored, a rapid malaria test done and haemoglobin measured. Specimens are not collected from children admitted electively or those with surgical problems, trauma, acute burns or non-infectious neonatal problems. Children admitted with suspected sepsis have a blood culture done and those with suspected meningitis a blood culture and lumbar puncture. For those admitted with clinical pneumonia (table 1) or a danger sign or focal chest signs, a blood culture, NP swab and chest X- ray will be performed and pleural fluid or lung aspirate obtained as clinically indicated. For those with clinical pneumonia treated as an outpatient a NP specimen is taken. Other investigations are done according to clinical judgment. X-rays are performed and interpreted according to WHO recommendations. Two readings of each radiograph are undertaken by independent readers and readings discordant for end-point consolidation are resolved by a third reader. All readers are calibrated to the WHO standard for diagnosis of radiological pneumonia with consolidation before reading radiographs.

**Nasopharyngeal pneumococcal carriage**

NP flocked swabs are collected using recommended techniques and placed in media and transported to the laboratory. NP specimens are labeled with pre-printed labels with random laboratory numbers. Thus, laboratory staff are masked to the group allocation of patients. A 10µl loop of vortexed specimen is inoculated onto blood agar with 5% gentamicin and incubated in CO_2_. If alpha-haemolytic colonies are identified, the entire growth is swept from the plate and suspended in sterile saline. After adjustment of turbidity the suspension is serotyped using a latex agglutination technique to detect the presence of potentially multiple pneumococcal serotypes. The results are recorded in laboratory logbooks and entered into the trial database.

Standardisation and quality control for the collection of NP specimens by nursing and field staff includes frequent refresher training and supervision. Internal quality control on the serotyping of NP specimens consists of repeat serotyping of a random 10% sample of positive NP specimens by a blinded second operator. External quality control is conducted by blinded serotyping by Basse laboratory staff of spiked NP specimens. Further quality control is achieved by comparison of Basse laboratory results with selected positive specimens analyzed by molecular microarray serotyping at BUGS Bioscience, St George’s University of London.

**Radiological pneumonia**

The clinical procedures to ascertain cases of radiological pneumonia are described in the ‘Clinical endpoints’ section. Radiographic images are de-identified and blindly interpreted by two independent readers using the WHO standard for radiological pneumonia in children. Discordant readings for end-point consolidation or pleural effusion are resolved by a third reader. All readers are calibrated to the WHO standard with high levels of agreement on end-point consolidation with blinded samples of the WHO standard set of radiographs (kappa statistic >0.8) required before reading radiographs.

**Invasive pneumococcal disease**

The clinical procedures to ascertain cases of IPD are described in the ‘Clinical endpoints’ section. Blood is collected using sterile technique and inoculated into culture bottles (Bactec Peds Plus). An automated system (Bactec 9050, Becton Dickinson; BacT Alert) is used for blood cultures. Bottles that signal positive will be sub-cultured onto blood agar, chocolate agar, and McConkey agar. Bottles which fail to signal within 5 days will be considered negative. Isolates grown will be identified using conventional microbiological techniques and biochemical tests (API, Biomerieux). Other sterile site samples will be processed using standardized techniques.^27^ *S. pneumoniae* will be identified by colony morphology,

**Supplementary table 1.** **Clinical definitions for suspected pneumonia, septicaemia, and meningitis**

| **Suspected pneumonia** | Suspected pneumonia is defined if there is a history of cough or difficulty breathing of less than 14 days’ duration, accompanied by one or more of:  1. Raised respiratory rate for age^*^  Criteria for nurse  screening for clinical pneumonia  2. Lower chest wall indrawing  3. Oxygen saturation less than 93%  4. Nasal flaring  5. Grunting  6. Stridor  7. Gasping  Criteria for admission surveillance diagnosis of clinical pneumonia  8. Head nodding  9. History of convulsions (not a febrile convulsion)  10. Inability to sit or feed  11. Vomiting everything  12. Lethargy  13. Impaired consciousness  14. Dullness to percussion detected by a clinician  15. Bronchial breathing detected by a clinician  16. Coarse crackles detected by a clinician |
| --- | --- |
| **Suspected meningitis** | Suspected meningitis will be defined according to clinical judgement and is to be considered if any of the following are present:  1. Neck stiffness  2. Impaired consciousness^‡^  3. Prostration^§^  4. History of convulsion  5. Bulging fontanelle |
| **Suspected septicaemia** | Suspected septicaemia will be defined as one or more of:  1. Clinician diagnosis of focal sepsis (including but not limited to: septic arthritis, osteomyelitis, endocarditis, peritonitis, liver abscess, soft tissue abscess, cellulitis)  2. Axillary temperature is <36°C or ≥38°C in a child admitted, or being admitted with no obvious cause of fever  3. For a patient admitted, or being admitted, the clinical impression is of severe malnutrition^\|\|^ |

Cough or difficulty breathing accompanied by one or more of the signs numbered 1, 2, 3 is used to define clinical pneumonia at nurse screening.

Cough or difficulty breathing accompanied by one or more of the signs numbered 1 - 8 is used to define clinical pneumonia as the surveillance diagnosis by nurses in outlying clinics and doctors in Basse and Bansang.

*Raised respiratory rate for age is defined as greater than or equal to 60 breaths per minute for children less than 60 days of age, greater than or equal to 50 breaths per minute for children aged at least 2 months but less than 12 months, and as greater than or equal to 40 breaths per minute for children at least 12 months but less than 60 months. If the respiratory rate is raised on the first measurement it will be measured again after at least 5 minutes - and deemed raised if still greater than the given age-specific threshold.

‡Impaired consciousness is defined as V, P, or U on the AVPU score, where A is if the patient is alert, V if responsive to verbal stimulus, P if responsive to pain stimulus, and U if unresponsive.

§Prostration is defined as inability to feed, or to remain in a seated position in a child otherwise able to do so.

^||^Severe malnutrition is defined according to the WHO definition.

**Supplementary table 2. Guideline for investigation of patients**

1. Patients admitted with any acute medical problem will have a blood culture taken, a plasma aliquot stored, a rapid malaria test done and haemoglobin measured. Samples will not be collected from children admitted electively or those with surgical problems, trauma, acute burns or non-infectious neonatal problems.
2. Patients with suspected meningitis will have a lumbar puncture and chest X-ray done.
3. Patients with clinical pneumonia will have a NP swab. If admitted to hospital with clinical or suspected pneumonia, patients will have a chest X-ray, NP swab and blood culture.
4. Patients with suspected septicaemia without a focus will have a blood culture and chest X-ray done.
5. Patients with suspected septicaemia with a focus will have a blood culture only.
6. Lung aspirate will be considered if large peripheral consolidation is demonstrated by X-ray.
7. Other investigations, including pleural aspiration, may be considered according to clinical indication.

susceptibility to ethylhydrocupreine and, if susceptibility is equivocal, by bile solubility, and reaction with polyvalent antisera (Statens Serum Institut, Copenhagen, Denmark). Isolates classified as contaminants will include coagulase-negative staphylococcus, bacillus species, micrococcus species, and *Streptococcus viridans*.

**Hospitalisation**

Patients presenting to Basse and Bansang hospitals are reviewed by nursing staff and referred to research clinicians if admission to hospital is considered. The decision to admit to hospital overnight is made by the research clinician from Monday to Saturday from 0800 to 2000 and by MoH nursing staff if patients present overnight between 2000 and 0800 or on Sundays. Study nurses screen and evaluate all unwell children presenting to the nine outlying health facilities from 0800 to 2000. The decision to admit overnight is made by the study staff and approved by the MoH Officer-in-Charge. MoH staff evaluate and admit patients presenting overnight between 2000 and 0800. Admission overnight is recorded in the study EMR.

**Mortality**

Deaths of patients presenting unwell to health facilities in the study area are recorded in the study EMR with identifying and residential details and linked to the HDSS population list. Deaths detected by study staff at follow-up at EPI clinics or at home visits are recorded in the study database with identifying and residential details and linked to the HDSS population list. Deaths detected by HDSS staff at 4-monthly household enumerations are recorded in the HDSS database, with identifying and residential details.

**Supplementary table 3. Disease endpoints in children aged 0 – 59 months**

| Safety endpoint | Year | Alternative schedule | | | Standard schedule | | | Adj. incidence rate ratio (95% CI) |
| --- | --- | --- | --- | --- | --- | --- | --- | --- |
|  |  | Number | Person-  years | Incidence  (95% CI) | Number | Person-  years | Incidence  (95% CI) |  |
| VT **Spn* IPD | Year 1 |  |  |  |  |  |  |  |
|  | Year 2 |  |  |  |  |  |  |  |
|  | Year 3 |  |  |  |  |  |  |  |
|  | Year 4 |  |  |  |  |  |  |  |
|  | Year 1-4 |  |  |  |  |  |  |  |
| Non-VT  *Spn* IPD | Year 1 |  |  |  |  |  |  |  |
|  | Year 2 |  |  |  |  |  |  |  |
|  | Year 3 |  |  |  |  |  |  |  |
|  | Year 4 |  |  |  |  |  |  |  |
|  | Year 1-4 |  |  |  |  |  |  |  |
| Radiological  pneumonia | Year 1 |  |  |  |  |  |  |  |
|  | Year 2 |  |  |  |  |  |  |  |
|  | Year 3 |  |  |  |  |  |  |  |
|  | Year 4 |  |  |  |  |  |  |  |
|  | Year 1-4 |  |  |  |  |  |  |  |
| Clinical  pneumonia | Year 1 |  |  |  |  |  |  |  |
|  | Year 2 |  |  |  |  |  |  |  |
|  | Year 3 |  |  |  |  |  |  |  |
|  | Year 4 |  |  |  |  |  |  |  |
|  | Year 1-4 |  |  |  |  |  |  |  |
| ††Clinical  pneumonia  NP VT *Spn* | Year 1 |  |  |  |  |  |  |  |
|  | Year 2 |  |  |  |  |  |  |  |
|  | Year 3 |  |  |  |  |  |  |  |
|  | Year 4 |  |  |  |  |  |  |  |
|  | Year 1-4 |  |  |  |  |  |  |  |
| Hospitalisation | Year 1 |  |  |  |  |  |  |  |
|  | Year 2 |  |  |  |  |  |  |  |
|  | Year 3 |  |  |  |  |  |  |  |
|  | Year 4 |  |  |  |  |  |  |  |
|  | Year 1-4 |  |  |  |  |  |  |  |
| Mortality | Year 1 |  |  |  |  |  |  |  |
|  | Year 2 |  |  |  |  |  |  |  |
|  | Year 3 |  |  |  |  |  |  |  |
|  | Year 4 |  |  |  |  |  |  |  |
|  | Year 1-4 |  |  |  |  |  |  |  |
| Non-*Spn* invasive bacterial disease | Year 1 |  |  |  |  |  |  |  |
|  | Year 2 |  |  |  |  |  |  |  |
|  | Year 3 |  |  |  |  |  |  |  |
|  | Year 4 |  |  |  |  |  |  |  |
|  | Year 1-4 |  |  |  |  |  |  |  |
| Diarrhoea | Year 1 |  |  |  |  |  |  |  |
|  | Year 2 |  |  |  |  |  |  |  |
|  | Year 3 |  |  |  |  |  |  |  |
|  | Year 4 |  |  |  |  |  |  |  |
|  | Year 1-4 |  |  |  |  |  |  |  |

*Spn* – *S. pneumoniae.* ††Clinical pneumonia with NP VT *Spn* carriage in children aged 2-260 weeks.

**Supplementary table 4. Individual-level baseline characteristics of all infants enrolled at EPI clinics**

| **Characteristics** | **Group** | |
| --- | --- | --- |
| **Individual-level** | **X** | **Y** |
| Number of eligible infants | n=x | n=y |
| Number of enrolled infants | n=x | n=y |
| Age at enrolment (weeks), n | n=x | n=y |
| median (IQR) | x.x (x.x-x.x) | y.y(y.y-y.y) |
| Infants sex, n | n=x | n=y |
| female, n (%) | x (x.x%) | y (y.y%) |
| Mother's age (years), n | n=x | n=y |
| median (IQR) | x (x-x) | y (y-y) |
| No. household members, n | n=x | n=y |
| median (IQR) | x (x-x) | y (y-y) |
| No. household children aged <15 years, n | n=x | n=y |
| median (IQR) | x (x-x) | y (y-y) |
| Age dose 1 PCV (weeks) , n (dose given) | n=x | n=y |
| median (IQR) | x (x-x) | y (y-y) |
| Age dose 2 Penta [alternative sch.]; dose 2 PCV [standard sch.]  (weeks), n (dose given) | n=x | n=y |
| median (IQR) | x (x-x) | y (y-y) |
| Age dose 3 Penta [alternative sch.]; dose 3 PCV [standard sch.]  (weeks) , n (dose given) | n=x | n=y |
| median (IQR) | x (x-x) | y (y-y) |
| Age dose 2 PCV [alternative sch.]; dose 1 measles [standard sch.]  (weeks) , n (dose given) | n=x | n=y |
| median (IQR) | x (x-x) | y (y-y) |

**Supplementary table 5. Individual-level baseline characteristics of infants enrolled at EPI clinics, by year**

| **Characteristics** | **Year 1** | | **Year 2** | | **Year 3** | | **Year 4** | |
| --- | --- | --- | --- | --- | --- | --- | --- | --- |
| **Individual level** | **X** | **Y** | **X** | **Y** | **X** | **Y** | **X** | **Y** |
| Number of eligible infants | n=x | n=y | n=x | n=y | n=x | n=y | n=x | n=y |
| Number of enrolled infants | n=x | n=y | n=x | n=y | n=x | n=y | n=x | n=y |
| Age at enrolment (weeks), n | n=x | n=y | n=x | n=y | n=x | n=y | n=x | n=y |
| median (IQR) | x.x (x.x-x.x) | y.y (y.y-y.y) | x.x (x.x-x.x) | y.y (y.y-y.y) | x.x (x.x-x.x) | y.y (y.y-y.y) | x.x (x.x-x.x) | y.y (y.y-y.y) |
| Infants sex, n | n=x | n=y | n=x | n=y | n=x | n=y | n=x | n=y |
| female, n (%) | x (x.x%) | y (y.y%) | x (x.x%) | y (y.y%) | x (x.x%) | y (y.y%) | x (x.x%) | y (y.y%) |
| Mother's age (years), n | n=x | n=y | n=x | n=y | n=x | n=y | n=x | n=y |
| median (IQR) | x (x-x) | y (y-y) | x (x-x) | y (y-y) | x (x-x) | y (y-y) | x (x-x) | y (y-y) |
| No. household members, n | n=x | n=y | n=x | n=y | n=x | n=y | n=x | n=y |
| median (IQR) | x (x-x) | y (y-y) | x (x-x) | y (y-y) | x (x-x) | y (y-y) | x (x-x) | y (y-y) |
| No. household children aged <15 years, n | n=x | n=y | n=x | n=y | n=x | n=y | n=x | n=y |
| median (IQR) | x (x-x) | y (y-y) | x (x-x) | y (y-y) | x (x-x) | y (y-y) | x (x-x) | y (y-y) |
| Age dose 1 PCV (weeks) , n | n=x | n=y | n=x | n=y | n=x | n=y | n=x | n=y |
| median (IQR) | x (x-x) | y (y-y) | x (x-x) | y (y-y) | x (x-x) | y (y-y) | h | y (y-y) |
| Age dose 2 Penta [alternative sch.]; dose 2 PCV  [standard sch.] (weeks), n | n=x | n=y | n=x | n=y | n=x | n=y | n=x | n=y |
| median (IQR) | x (x-x) | y (y-y) | x (x-x) | y (y-y) | x (x-x) | y (y-y) | x (x-x) | y (y-y) |
| Age dose 3 Penta [alternative sch.]; dose 3 PCV  [standard sch.] (weeks), n | n=x | n=y | n=x | n=y | n=x | n=y | n=x | n=y |
| median (IQR) | x (x-x) | y (y-y) | x (x-x) | y (y-y) | x (x-x) | y (y-y) | x (x-x) | y (y-y) |
| Age dose 2 PCV [alternative sch.]; dose 1 measles  [standard sch.] (weeks), n | n=x | n=y | n=x | n=y | n=x | n=y | n=x | n=y |
| median (IQR) | x (x-x) | y (y-y) | x (x-x) | y (y-y) | x (x-x) | y (y-y) | x (x-x) | y (y-y) |
| Age dose 2 measles (weeks), n | n=x | n=y | n=x | n=y | n=x | n=y | n=x | n=y |
| median (IQR) | x (x-x) | y (y-y) | x (x-x) | y (y-y) | x (x-x) | y (y-y) | x (x-x) | y (y-y) |

**Supplementary table 6. Cluster-level baseline characteristics of infants enrolled at EPI clinics**

| **Characteristics** | **Group** | |
| --- | --- | --- |
| **Cluster-level** | **X** | **Y** |
| Number of eligible infants | n=x | n=y |
| Number of enrolled infants | n=x | n=y |
| Age at enrolment (weeks) (no./cluster), n | n=x | n=y |
| median of cluster medians (IQR) | x.x (x.x-x.x) | y.y (y.y-y.y) |
| min-max | n.n-n.n | y.y-y.y |
| All residents (no./cluster), n | n=n | n=y |
| median no./cluster (IQR) | n (n-n) | y (y-y) |
| min-max | n-n | y-y |
| Households with enrolled children (no./cluster), n | n=x | n=y |
| median no./cluster (IQR) | n (n-n) | y (y-y) |
| min-max | n-n | y-y |
| Received 1st dose PCV age <=10 weeks, n/N (%) | n/N (n.n%) | y/y (y.y%) |
| median of percentages (IQR) | n% (n%-n%) | y% (y%-y%) |
| min-max | n%-n% | y%-y% |
| Received 3rd dose Penta age <=22 weeks, n/N (%) | n/N (n.n%) | y/y (y.y%) |
| median of percentages (IQR) | n% (n%-n%) | y% (y%-y%) |
| min-max | x%-x% | y%-y% |
| Received 1st dose Measles age <=48 weeks, n/N (%) | x/x (x.x%) | y/y (y.y%) |
| median of percentages (IQR) | x% (x%-x%) | y% (y%-y%) |
| min-max | x%-x% | y%-y% |
| No. internal inmigrations, n | x | y |
| median no./cluster (IQR) | x (x-x) | y (y-y) |
| min-max | x-x | y-y |
| No. outmigrations, n | x | y |
| median no./cluster (IQR) | x (x-x) | y (y-y) |
| min-max | x-x | y-y |
| No. external inmigrations, n | x | y |
| median no./cluster (IQR) | x (x-x) | y (y-y) |
| min-max | x-x | y-y |

**Supplementary figure 1. Monthly number of infants screened, eligible, and enrolled at EPI clinics**


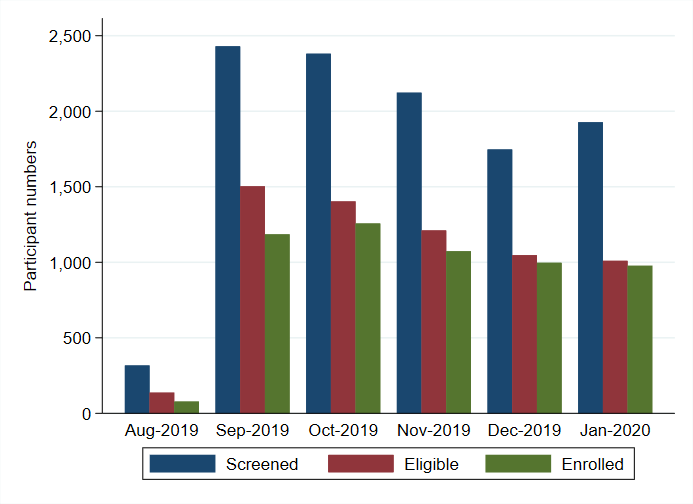


**Supplementary figure 2. Cumulative number of infants screened, eligible, and enrolled at EPI clinics**


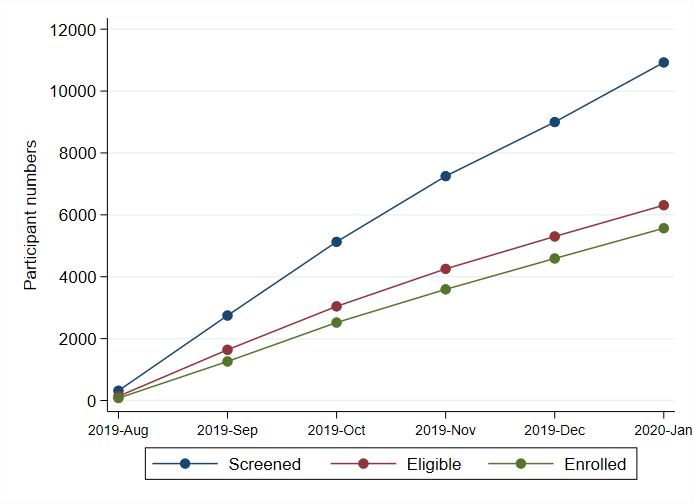


**Supplementary figure 3. Flowchart of patient presentation and eligibility for measurement of the primary endpoint at health facilities**

Eligible for clinical pneumonia screening in:

Year 1 n=x (%)

Clinical pneumonia screening n=x (%)

Screening not performed/recorded n=x (%)

Year 2 n=x (%)

Clinical pneumonia screening n=x (%)

Screening not performed/recorded n=x (%)

Year 3 n=x (%)

Clinical pneumonia screening n=x (%)

Screening not performed/recorded n=x (%)

Year 4 n=x (%)

Clinical pneumonia screening n=x (%)

Screening not performed/recorded n=x (%)

Years 1-4 n=x (%)

Clinical pneumonia screening n=x (%)

Screening not performed/recorded n=x (%)

Eligible for clinical pneumonia screening in:

Year 1 n=x (%)

Clinical pneumonia screening n=x (%)

Screening not performed/recorded n=x (%)

Year 2 n=x (%)

Clinical pneumonia screening n=x (%)

Screening not performed/recorded n=x (%)

Year 3 n=x (%)

Clinical pneumonia screening n=x (%)

Screening not performed/recorded n=x (%)

Year 4 n=x (%)

Clinical pneumonia screening n=x (%)

Screening not performed/recorded n=x (%)

Years 1-4 n=x (%)

Clinical pneumonia screening n=x (%)

Screening not performed/recorded n=x (%)

Presented at health facilities in Year 1 n=x

Year 2 n=x

Year 3 n=x

Year 4 n=x

Years 1-4 n=x

Presented at health facilities in Year 1 n=x

Year 2 n=x

Year 3 n=x

Year 4 n=x

Years 1-4 n=x

Geographic clusters in Basse and Fuladu West HDSSs (n=68)

Geographic clusters randomised (n=68)

Clusters allocated to X (n=35)

Residents aged 0-260 weeks in Year 1 n=x

Median no./cluster n=x (IQR)

Min-Max/cluster n=x

Residents aged 0-260 weeks in Year 2 n=x

Median no./cluster n=x (IQR)

Min-Max/cluster n=x

Residents aged 0-260 weeks in Year 3 n=x

Median no./cluster n=x (IQR)

Min-Max/cluster n=x

Residents aged 0-260 weeks in Year 4 n=x

Median no./cluster n=x (IQR)

Min-Max/cluster n=x

Clusters allocated to Y (n=33)

Residents aged 0-260 weeks in Year 1 n=y

Median no./cluster n=y (IQR)

Min-Max/cluster n=y

Residents aged 0-260 weeks in Year 2 n=x

Median no./cluster n=x (IQR)

Min-Max/cluster n=x

Residents aged 0-260 weeks in Year 3 n=x

Median no./cluster n=x (IQR)

Min-Max/cluster n=x

Residents aged 0-260 weeks in Year 4 n=x

Median no./cluster n=x (IQR)

Min-Max/cluster n=x

**Supplementary figure 4. Flowchart of patient investigation and results of primary endpoint surveillance at health facilities**

Geographic clusters in Basse and Fuladu West HDSSs (n=68)

Clusters allocated to X (n=35)

Residents aged 0-260 weeks in Year 1 n=x

Clinical pneumonia/screened n/N

NPS collected n=x

NPS cultured n=x

NPS pneumococcus detected n=x

Serotyping result n=x

One serotype n=x

Multiple serotypes n=x

Incomplete serotypes n/N

Clusters allocated to Y (n=35)

Residents aged 0-260 weeks in Year 1 n=y

Clinical pneumonia/screened n/N

NPS collected n=y

NPS cultured n=y

NPS pneumococcus detected n=y

Serotyping result n=y

One serotype n=y

Multiple serotypes n=y

Incomplete serotypes n/N

Residents aged 0-260 weeks in Year 2 n=x

Clinical pneumonia/screened n/N

NPS collected n=x

NPS cultured n=x

NPS pneumococcus detected n=x

Serotyping result n=x

One serotype n=x

Multiple serotypes n=x

Incomplete serotypes n/N

Residents aged 0-260 weeks in Year 2 n=y

Clinical pneumonia/screened n/N

NPS collected n=y

NPS cultured n=y

NPS pneumococcus detected n=y

Serotyping result n=y

One serotype n=y

Multiple serotypes n=y

Incomplete serotypes n/N

Residents aged 0-260 weeks in Year 3 n=y

Clinical pneumonia/screened n/N

NPS collected n=y

NPS cultured n=y

NPS pneumococcus detected n=y

Serotyping result n=y

One serotype n=y

Multiple serotypes n=y

Incomplete serotypes n/N

Residents aged 0-260 weeks in Year 3 n=x

Clinical pneumonia/screened n/N

NPS collected n=x

NPS cultured n=x

NPS pneumococcus detected n=x

Serotyping result n=x

One serotype n=x

Multiple serotypes n=x

Incomplete serotypes n/N

Residents aged 0-260 weeks in Year 4 n=y

Clinical pneumonia/screened n/N

NPS collected n=y

NPS cultured n=y

NPS pneumococcus detected n=y

Serotyping result n=y

One serotype n=y

Multiple serotypes n=y

Incomplete serotypes n/N

Residents aged 0-260 weeks in Year 4 n=x

Clinical pneumonia/screened n/N

NPS collected n=x

NPS cultured n=x

NPS pneumococcus detected n=x

Serotyping result n=x

One serotype n=x

Multiple serotypes n=x

Incomplete serotypes n/N

**Supplementary table 7. Baseline information on clinical endpoint surveillance at health facilities, at the individual-level**

|  | | **Group** | |
| --- | --- | --- | --- |
|  | | **X** | **Y** |
| **Number resident children aged 0-260 weeks**, Year 1 | | N=x | N=y |
| Year 2 | | N=x | N=y |
| Year 3 | | N=x | N=y |
| Year 4 | | N=x | N=y |
| Year 1-4 | | N=x | N=y |
| **Health facility registrations** | | **Aggregate** | |
| Age (weeks), n – median (IQR), Year 1 | | n=x – x (x-x) | |
| Year 2 | | n=x – x (x-x) | |
| Year 3 | | n=x – x (x-x) | |
| Year 4 | | n=x – x (x-x) | |
| Year 1-4 | | n=x – x (x-x) | |
| Sex, female n/N (%), Year 1 | | n/N (%) | |
| Year 2 | | n/N (%) | |
| Year 3 | | n/N (%) | |
| Year 4 | | n/N (%) | |
| Year 1-4 | | n/N (%) | |
| **Patients with clinical pneumonia** | | **X** | **Y** |
| Age (weeks), n – median (IQR), Year 1 | | n=x – x (x-x) | n=y – y (y-y) |
| Year 2 | | n=x – x (x-x) | n=y – y (y-y) |
| Year 3 | | n=x – x (x-x) | n=y – y (y-y) |
| Year 4 | | n=x – x (x-x) | n=y – y (y-y) |
| Year 1-4 | | n=x – x (x-x) | n=y – y (y-y) |
| Sex, female n/N (%), Year 1 | | n/N (%) | n/N (%) |
| Year 2 | | n/N (%) | n/N (%) |
| Year 3 | | n/N (%) | n/N (%) |
| Year 4 | | n/N (%) | n/N (%) |
| Year 1-4 | | n/N (%) | n/N (%) |
| Admitted to hospital n/N (%), Year 1 | | n/N (%) | n/N (%) |
| Year 2 | | n/N (%) | n/N (%) |
| Year 3 | | n/N (%) | n/N (%) |
| Year 4 | | n/N (%) | n/N (%) |
| Year 1-4 | | n/N (%) | n/N (%) |
| **Admitted children** | |  |  |
| Weight-for-height z-score <-3, N - n/N (%), Year 1 | | N - x/x (%) | N - y/y (%) |
| Year 2 | | N - x/x (%) | N - y/y (%) |
| Year 3 | | N - x/x (%) | N - y/y (%) |
| Year 4 | | N - x/x (%) | N - y/y (%) |
| Year 1-4 | | N – x/x (%) | N – y/y (%) |
| Malaria RDT positive, N - n/N (%), Year 1 | | N - x/x (%) | N - y/y (%) |
| Year 2 | | N - x/x (%) | N - y/y (%) |
| Year 3 | | N - x/x (%) | N - y/y (%) |
| Year 4 | | N - x/x (%) | N - y/y (%) |
| Year 1-4 | | N – x/x (%) | N – y/y (%) |
| Haemoglobin, N – n, median (IQR), Year 1 | | N - n, x (x-x) | N – n, y (y-y) |
| Year 2 | | N - n, x (x-x) | N – n, y (y-y) |
| Year 3 | | N - n, x (x-x) | N – n, y (y-y) |
| Year 4 | | N - n, x (x-x) | N – n, y (y-y) |
| Year 1-4 | | N - n, x (x-x) | N – n, y (y-y) |
| Clinical pneumonia and  radiograph, N – n/N (%), | Year 1 | N - x/x (%) | N - x/x (%) |
|  | Year 2 | N - x/x (%) | N - x/x (%) |
|  | Year 3 | N - x/x (%) | N - x/x (%) |
|  | Year 4 | N - x/x (%) | N - x/x (%) |
|  | Year 1-4 | N - x/x (%) | N - x/x (%) |
| Non-pneumococcal invasive  bacterial disease, N – n/N (%), | Year 1 | N - x/x (%) | N - x/x (%) |
|  | Year 2 | N - x/x (%) | N - x/x (%) |
|  | Year 3 | N - x/x (%) | N - x/x (%) |
|  | Year 4 | N - x/x (%) | N - x/x (%) |
|  | Year 1-4 | N - x/x (%) | N - x/x (%) |
| Diarrhoeal disease, N – n/N (%), | Year 1 | N - x/x (%) | N - x/x (%) |
|  | Year 2 | N - x/x (%) | N - x/x (%) |
|  | Year 3 | N - x/x (%) | N - x/x (%) |
|  | Year 4 | N - x/x (%) | N - x/x (%) |
|  | Year 1-4 | N - x/x (%) | N - x/x (%) |

**Supplementary table 8. Baseline information on endpoint surveillance at health facilities, at the cluster level**

| **Cluster level (calculated by cluster, then summarized)** | | **Aggregate** | |
| --- | --- | --- | --- |
| **Number resident children aged 0-260 weeks**, Year 1 | | n | |
| Year 2 | | n | |
| Year 3 | | n | |
| Year 4 | | n | |
| Year 1-4 | | n | |
| **Health facility registrations** | |  | |
| Age group | |  | |
| 0-28 days, N – median no./cluster (IQR), Year 1 | | N – n (a-b) | |
| Year 2 | | N – n (a-b) | |
| Year 3 | | N – n (a-b) | |
| Year 4 | | N – n (a-b) | |
| Year 1-4 | | N – n (a-b) | |
| 4-52 weeks, N – median no./cluster (IQR), Year 1 | | N – n (a-b) | |
| Year 2 | | N – n (a-b) | |
| Year 3 | | N – n (a-b) | |
| Year 4 | | N – n (a-b) | |
| Year 1-4 | | N – n (a-b) | |
| 52-260 weeks, N – median no./cluster (IQR), Year 1 | | N – n (a-b) | |
| Year 2 | | N – n (a-b) | |
| Year 3 | | N – n (a-b) | |
| Year 4 | | N – n (a-b) | |
| Year 1-4 | | N – n (a-b) | |
| Sex, female, N - weighted proportion/cluster (%), Year 1 | | N – x (%) | |
| Year 2 | | N - x (%) | |
| Year 3 | | N - x (%) | |
| Year 4 | | N - x (%) | |
| Year 1-4 | | N - x (%) | |
| **Patients with clinical pneumonia** | | **X** | **Y** |
| Age group | |  |  |
| 0-28 days, N – median no./cluster (IQR), Year 1 | | N – n (a-b) | N – n (a-b) |
| Year 2 | | N – n (a-b) | N – n (a-b) |
| Year 3 | | N – n (a-b) | N – n (a-b) |
| Year 4 | | N – n (a-b) | N – n (a-b) |
| Year 1-4 | | N – n (a-b) | N – n (a-b) |
| 4-52 weeks, N – median no./cluster (IQR), Year 1 | | N – n (a-b) | N – n (a-b) |
| Year 2 | | N – n (a-b) | N – n (a-b) |
| Year 3 | | N – n (a-b) | N – n (a-b) |
| Year 4 | | N – n (a-b) | N – n (a-b) |
| Year 1-4 | | N – n (a-b) | N – n (a-b) |
| 52-260 weeks, N – median no./cluster (IQR), Year 1 | | N – n (a-b) | N – n (a-b) |
| Year 2 | | N – n (a-b) | N – n (a-b) |
| Year 3 | | N – n (a-b) | N – n (a-b) |
| Year 4 | | N – n (a-b) | N – n (a-b) |
| Year 1-4 | | N – n (a-b) | N – n (a-b) |
| Sex, female, N – weighted proportion/cluster (%), Year 1 | | n/N (%) | n/N (%) |
| Year 2 | | n/N (%) | n/N (%) |
| Year 3 | | n/N (%) | n/N (%) |
| Year 4 | | n/N (%) | n/N (%) |
| Year 1-4 | | n/N (%) | n/N (%) |
| Admitted to hospital n/N (%), Year 1 | | n/N (%) | n/N (%) |
| Year 2 | | n/N (%) | n/N (%) |
| Year 3 | | n/N (%) | n/N (%) |
| Year 4 | | n/N (%) | n/N (%) |
| Year 1-4 | | n/N (%) | n/N (%) |
| **Admitted children** | |  |  |
| Weight-for-height z-score <-3, n/N -weighted proportion/cluster (%) | Year 1 | n/N – x (%) | n/N – y (%) |
|  | Year 2 | n/N – x (%) | n/N – y (%) |
| Year 3 | | n/N – x (%) | n/N – y (%) |
| Year 4 | | n/N – x (%) | n/N – y (%) |
| Year 1-4 | | n/N – x (%) | n/N – y (%) |
| Malaria RDT positive, n/N -  weighted proportion/cluster (%) | Year 1 | n/N – x (%) | n/N – y (%) |
|  | Year 2 | n/N – x (%) | n/N – y (%) |
| Year 3 | | n/N – x (%) | n/N – y (%) |
| Year 4 | | n/N – x (%) | n/N – y (%) |
| Year 1-4 | | n/N – x (%) | n/N – y (%) |
| Haemoglobin, n/N -  median/cluster medians (IQR) | Year 1 | n/N – x (a-b) | n/N - y (a-b) |
|  | Year 2 | n/N – x (a-b) | n/N - y (a-b) |
| Year 3 | | n/N – x (a-b) | n/N - y (a-b) |
| Year 4 | | n/N – x (a-b) | n/N - y (a-b) |
